# Genomic analysis reveals key aspects of prokaryotic symbiosis in the phototrophic consortium “*Chlorochromatium aggregatum*”

**DOI:** 10.1186/gb-2013-14-11-r127

**Published:** 2013-11-22

**Authors:** Zhenfeng Liu, Johannes Müller, Tao Li, Richard M Alvey, Kajetan Vogl, Niels-Ulrik Frigaard, Nathan C Rockwell, Eric S Boyd, Lynn P Tomsho, Stephan C Schuster, Petra Henke, Manfred Rohde, Jörg Overmann, Donald A Bryant

**Affiliations:** 1 Department of Biochemistry and Molecular Biology, The Pennsylvania State University, University Park, PA 16802, USA; 2 Leibniz-Institut DSMZ-Deutsche Sammlung von Mikroorganismen und Zellkulturen GmbH, Inhoffenstraße 7B, 38124, Braunschweig, Germany; 3 Section for Marine Biology, Department of Biology, University of Copenhagen, Strandpromenaden 5 3000, Helsingør, Denmark; 4 Department of Molecular and Cellular Biology, University of California, Davis, CA 95616, USA; 5 Department of Microbiology, Montana State University, Bozeman, MT 59717, USA; 6 Helmholtz-Zentrum für Infektionsforschung, 38124 Braunschweig, Germany; 7 Current address: Department of Biological Sciences, University of Southern California, Los Angeles, CA 90089, USA; 8 Current address: Algal Genomics Research Group, Institute of Hydrobiology, Chinese Academy of Sciences, Wuhan, Hubei 430072, China; 9 Current address: Department of Biology, Chaminade University, Honolulu, HI 96816, USA

## Abstract

**Background:**

‘*Chlorochromatium aggregatum*’ is a phototrophic consortium, a symbiosis that may represent the highest degree of mutual interdependence between two unrelated bacteria not associated with a eukaryotic host. ‘*Chlorochromatium aggregatum*’ is a motile, barrel-shaped aggregate formed from a single cell of ‘*Candidatus* Symbiobacter mobilis”, a polarly flagellated, non-pigmented, heterotrophic bacterium, which is surrounded by approximately 15 epibiont cells of *Chlorobium chlorochromatii,* a non-motile photolithoautotrophic green sulfur bacterium.

**Results:**

We analyzed the complete genome sequences of both organisms to understand the basis for this symbiosis. *Chl. chlorochromatii* has acquired relatively few symbiosis-specific genes; most acquired genes are predicted to modify the cell wall or function in cell-cell adhesion. In striking contrast, ‘*Ca.* S. mobilis’ appears to have undergone massive gene loss, is probably no longer capable of independent growth, and thus may only reproduce when consortia divide. A detailed model for the energetic and metabolic bases of the dependency of ‘*Ca*. S. mobilis’ on *Chl. chlorochromatii* is described.

**Conclusions:**

Genomic analyses suggest that three types of interactions lead to a highly sophisticated relationship between these two organisms. Firstly, extensive metabolic exchange, involving carbon, nitrogen, and sulfur sources as well as vitamins, occurs from the epibiont to the central bacterium. Secondly, ‘*Ca*. S. mobilis’ can sense and move towards light and sulfide, resources that only directly benefit the epibiont. Thirdly, electron cycling mechanisms, particularly those mediated by quinones and potentially involving shared protonmotive force, could provide an important basis for energy exchange in this and other symbiotic relationships.

## Background

Symbiotic interactions between bacteria and eukaryotes are common and can be mutualistic (for example, between nitrogen-fixing *Rhizobium* spp. and legumes [1] or between sulfur-oxidizing *Gamma-* or *Epsilonbacteria* and marine invertebrates [2]) or parasitic (for example, bacterial pathogens and human hosts). Archaea and eukaryotes also form symbioses, which include the methanogens of arthropod, ruminant, and human digestive systems as well as the archaeal symbionts of sponges [3]. Symbioses involving only bacterial and/or archaeal partners are also known and may be more widespread than commonly recognized [4]. Mutualistic interactions involving nutrient exchange, waste removal, and stress protection are probably crucial to the maintenance of microbial biofilm communities and are well documented in syntrophic interactions involving hydrogen or formate transfer [5]. Other examples include chlorophototrophic mat communities of hot springs [6] and anaerobic methane-oxidizing communities of marine environments [7].

**Figure 1:**
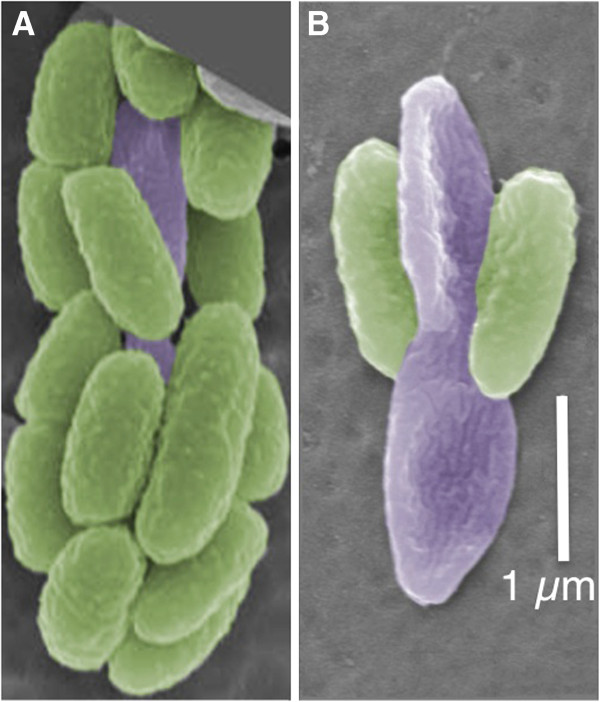
**Scanning electron micrographs of “*Chlorochromatium aggregatum*”. (A,B)** Epibionts are shown in false color green, central bacteria in false color purple. **(B)** The central rod is dividing, and most of the epibiont cells have dissociated from the consortium. Scale bar in **(B)** equals 1 μm.

The term ‘consortium’ originally described a close association of microbial cells in which two or more different microorganisms maintained an organized structure through permanent cell-to-cell contact [8]. Phototrophic consortia were first reported more than 100 years ago [9], and they may represent the highest degree of mutual interdependence between two unrelated bacteria not associated with a eukaryotic host [4,10-12]. Ten morphologically distinct types are known, and most are barrel-shaped, motile aggregates comprising two cell types: a central bacterium, which is a single, non-pigmented, and heterotrophic cell carrying a single polar flagellum; and approximately 15 to >40 epibionts, which are green- or brown-colored green sulfur bacteria (GSB) [10-12] (Figure 1; Figure S1 in Additional file 1). These consortia are motile and exhibit scotophobotaxis (‘fear of the dark’) as well as positive chemotaxis to sulfide, thiosulfate, 2-oxoglutarate and citrate [13].

Because of the availability of an enrichment culture isolated from Lake Dagow, Germany, *“Chlorochromatium aggregatum”* is the best-characterized phototrophic consortium [13]. The epibiont of *“Chlorochromatium aggregatum”, Chlorobium* (*Chl.*) *chlorochromatii* strain CaD3, is not obligately symbiotic. It has been isolated and grown axenically, and physiological characterization showed that *Chl. chlorochromatii* is similar to other free-living GSB isolates [14]. It is a non-motile, obligately anaerobic, photolithoautotrophic GSB that uses sulfide as the electron donor for photosynthetic CO_2_ and N_2_ fixation. The genome of *Chl. chlorochromatii* has been sequenced, and this enabled previous biochemical, transcriptomic and proteomic studies of *Chl. chlorochromatii*[15,16]*.* The central bacterium of *“Chlorochromatium aggregatum”*, hereafter denoted as “*Candidatus* Symbiobacter (*Ca.* S.) mobilis”, is a rod-shaped member of the Betaproteobacteria (Figure 1; Figure S1 in Additional file 1). It has a single polar flagellum [17] and is most similar to non-symbiotic bacteria of family Comamonadaceae [18]. All attempts to grow the central bacterium independently from the epibionts have failed. Phylogenetic analyses have shown that the epibionts and central bacteria of different types of consortia and lakes have polyphyletic origins [19-21]. To gain insights into the molecular basis for the symbiotic relationship in phototrophic consortia, we determined the complete genome sequence of “*Ca.* S. mobilis”, analyzed these genomes, and present here a description of *“Chlorochromatium aggregatum”*. Compared to free-living close relatives, “*Ca.* S. mobilis” has apparently undergone massive gene loss and is probably no longer capable of independent growth.

## Results and discussion

### The two genomes of “*Chlorochromatium aggregatum*”

**Figure 2:**
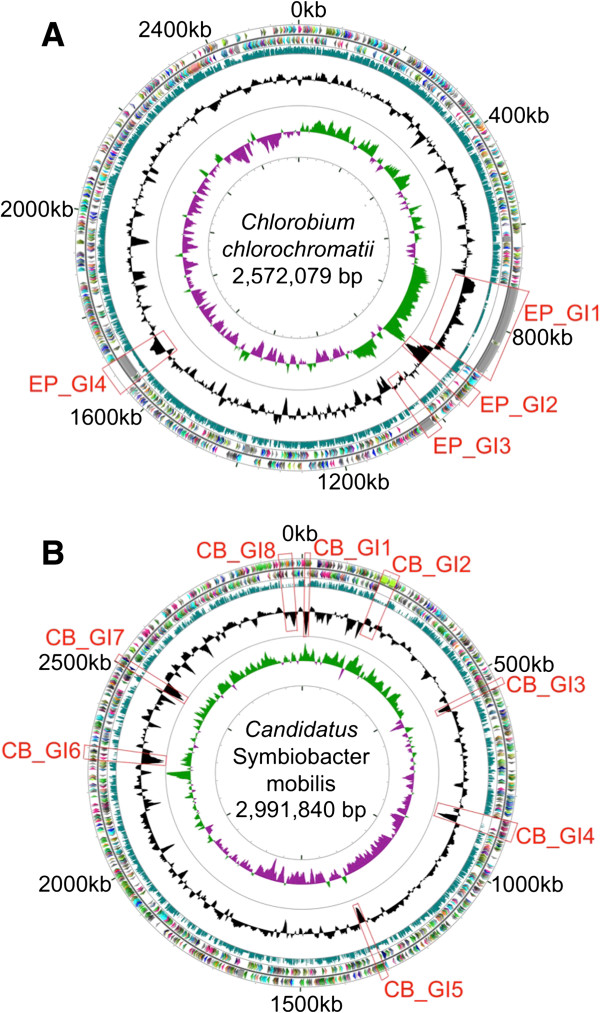
**Circular maps and genomic islands of the genomes of *Chl. chlorochromatii *(A) and “*Ca*. S. mobilis” (B).** From outside in, the circles represent open reading frames (ORFs) on the forward strand, ORFs on the reverse strand, BLASTP scores of ORFs against reference genomes, mol% G + C, and GC skew. Colors of ORFs represent COG (clusters of orthologous groups of proteins) categories. Mol% G + C is plotted using genome averages as baselines, which are 44.3% and 59.1% for the epibiont and the central bacterium, respectively. Genomic islands are marked by red boxes (see Materials and methods for identification of genomic islands). The reference genomes used in this study are: *Chlorobaculum parvum* NCIB 8327, *Chlorobaculum tepidum* ATCC 49652, *Chlorobium ferrooxidans* DSM 13031, *Chlorobium limicola* DSM 245, *Chlorobium phaeobacteroides* BS-1, *Chlorobium phaeobacteroides* DSM 266, *Chlorobium phaeovibrioides* DSM 265, *Chlorobium clathratiforme* DSM 5477, *Chlorobium luteolum* DSM 273, *Prosthecochloris aestuarii* DSM 271, and *Chloroherpeton thalassium* ATCC 35110 for *Chl. chlorochromatii*; *Acidovorax avenae citrulli* str. AAC00-1, *Rhodoferax ferrireducens* DSM 15236, *Alicycliphilus denitrificans* str. BC, *Comamonas testosteroni* str. CNB-2, *Delftia acidovorans* str. SPH-1, *Polaromonas naphthalenivorans* str. CJ2, *Variovorax paradoxus* str. EPS, and *Verminephrobacter eiseniae* str. EF01-2 for “*Ca*. S. mobilis.”

The *Chl. chlorochromatii* genome (GenBank accession number CP000108) is a single circular DNA molecule of 2,572,079 bp with a G + C content of 44.3 mol%. It encodes 2,002 open reading frames (ORFs), one rRNA operon, and 45 tRNAs (Figure 2A). The size and gene content are very similar to those of 15 other GSB genomes [22]. Similar to the genomes of other GSB, the *Chl. chlorochromatii* genome encodes proteins for the photosynthetic apparatus, bacteriochlorophyll biosynthesis, sulfide oxidation, CO_2_ fixation via the reverse tricarboxylic acid (TCA) cycle, nitrogen fixation and all housekeeping genes of central metabolism and macromolecule biosynthesis [22-25]. Only 311 ORFs (15%), nearly all of which encode proteins with unidentified functions, have no homologs in genomes of other GSB, which are not known to be involved in phototrophic consortia (Table S1 in Additional file 2). These results are consistent with the observation that *Chl. chlorochromatii* is not obligately symbiotic and can grow independently as a photolithoautotroph [14]. Thus, only relatively minor changes in gene content were apparently required as the epibiont adapted to a symbiotic lifestyle.

**Figure 3:**
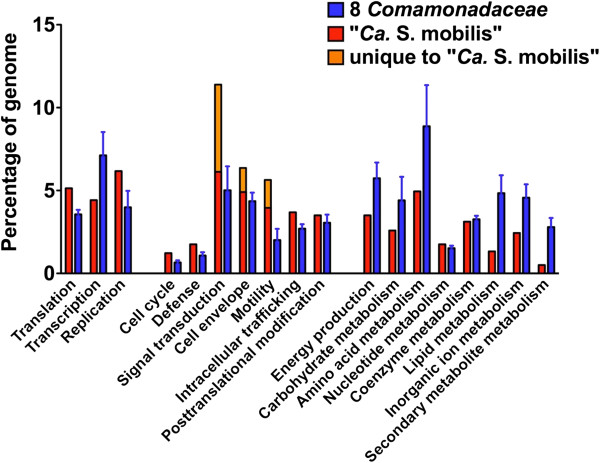
**Comparison of gene contents of “*Ca*. S. mobilis” and its relatives based on functional categories.** Percentages of genes for each COG category in the genomes are calculated for “*Ca*. S. mobilis” and its relatives based on COG assignment of genes provided by Integrated Microbial Genomes [28]. Averages and standard deviations of the percentages for the eight *Comamonadaceae* organisms listed in Figure 2 legend are shown.

In contrast, the genome of the “*Ca.* S. mobilis” differs dramatically from the genomes of eight close relatives with sequenced genomes (listed in the Figure 2 legend). The “*Ca.* S. mobilis” genome (GenBank accession number CP004885) is a single circular DNA molecule of 2,991,840 bp with G + C content of 59.1 mol%. It has two tandemly repeated rRNA operons [21], 44 tRNAs and 2,626 proteins (Figure 2B). The closest non-symbiotic relatives of “*Ca.* S. mobilis” from the family Comamonadaceae have much larger genomes (4.8 to 6.8 Mbp). “*Ca.* S. mobilis” apparently underwent substantial gene loss during its evolution, especially for genes involved in metabolism (Figure 3). Eight free-living members of the family Comamonadaceae, each representing a different genus, have a core genome of 1,284 genes, but 409 (32%) of these genes are missing from the “*Ca.* S. mobilis” genome (for a list of these missing genes, see Table S2 in Additional file 2). This degree of gene loss, which is common in exclusively symbiotic organisms [26,27], supports the experimental observation that “*Ca.* S. mobilis” is no longer capable of independent growth and now depends on its photoautotrophic partner for essential metabolites (see below).

On the other hand, “*Ca.* S. mobilis” has also acquired new genes, either through lateral gene transfer or gene duplication and subsequent diversification, that are not found in its close relatives. A comparison identified 1,055 “*Ca.* S. mobilis” genes without orthologs in any of eight free-living *Comamonadaceae* organisms; 444 (42%) of these genes have annotations other than ‘hypothetical protein’ (for a complete list, see Table S3 in Additional file 2). Genes involved in signal transduction (138), cell envelope biogenesis (38) and cell motility (44) are overrepresented. These gains and losses of genes resulted in different functional compositions for the genomes of “*Ca*. S. mobilis” and its relatives, especially in the categories mentioned above (Figure 3). Such differences presumably reflect adaptations of “*Ca.* S. mobilis” to an obligately symbiotic lifestyle, and they suggest that the major roles of “*Ca.* S. mobilis” are to sense the environment and to provide motility. The increased number of genes for cell wall and envelope biosynthesis is consistent with the importance of previously observed cell-to-cell contacts between the central rod and the epibionts via specialized cell-surface structures [17].

### Horizontal gene transfer

The genomes of “*Ca.* S. mobilis” and *Chl. chlorochromatii* were compared to search for potential horizontal gene transfers between these two partner organisms that are constantly in close contact. Thirteen pairs of genes in these two genomes are more similar to one another than to most if not all proteins in databases (Table S4 in Additional file 2); however, the functions of most could not be unambiguously assigned. Gene exchange (and gene transfer) between the two partners does not appear to have occurred frequently in this symbiosis.

The two genomes were also analyzed to identify genomic islands (GIs), which often harbor recently acquired or highly conserved genes. The identified GIs are marked in Figure 2 and their properties are summarized in Table 1 (GIs in *Chl. chlorochromatii* are denoted with the prefix ‘EP’; GIs in “*Ca.* S. mobilis” are denoted with the prefix ‘CB’). Four genomic islands were identified in *Chl. chlorochromatii*, and three contain unusually large proteins. These proteins are similar to hemagglutinin and outer membrane adhesin proteins of the RTX toxin family, which contain numerous, internally repeated, calcium-binding domains [29]. ORFs Cag_0614 and Cag_0616 in EP_GI-1 predict proteins of 36,805 and 20,646 amino acids, respectively. The former protein is larger than human titin (34,350 amino acids), often considered to be the largest known protein [30]. These two genes are transcribed, encode symbiosis-specific proteins, and have been hypothesized to stabilize contacts between the central bacterium and epibiont cells [15]. Smaller but related proteins, including the ones in EP_GI-3 (Cag_0738) and EP_GI-4 (Cag_1242), could play similar roles.

**Table 1:** **Properties of genomic islands in *Chl. chlorochromatii *** **and “ *Ca *. S. mobilis”**

**Genomic islands**	**Size (kb)**	**Number of genes**	**Transposase/integrase**	**Putative gene function**
EP_GI-1	175	3	No	Cell adhesion
EP_GI-2	34	36	Yes	Cell envelope biogenesis
EP_GI-3	36	8	No	Cell adhesion
EP_GI-4	49	4	No	Cell adhesion
CB_GI-1	12	10	Yes	Cell envelope biogenesis
CB_GI-2	37	4	No	Cell adhesion
CB_GI-3	11	9	No	Cell envelope biogenesis
CB_GI-4	48	45	Yes	CRISPR-associated and hypothetical proteins
CB_GI-5	16	17	No	Chemotaxis and regulation
CB_GI-6	44	33	Yes	Poorly defined genes
CB_GI-7	22	15	No	Cell envelope biosynthesis
CB_GI-8	31	27	Yes	Poorly defined genes

Eight GIs were identified in the “*Ca.* S. mobilis” genome (Figure 2B). The presence of transposases and integrases in most of them suggests that they were probably acquired by horizontal gene transfer. Genes found in the GIs of *Chl. chlorochromatii*, such as those involved in cell envelope biosynthesis and encoding haemagglutinin/adhesin-like proteins, were similarly found in CB_GI-1, CB_GI-2, CB_GI-3 and CB_GI-7. CB_GI-4 included mainly CRISPR-associated proteins and hypothetical proteins, while CB_GI-5 and CB_GI-7 contained mainly genes of unknown function. CB_GI-4 (Cenrod_1189-Cenrod_1205) encodes chemotaxis and regulatory proteins, and interestingly, this gene cluster is similar in both gene order and sequence to clusters found in several purple sulfur bacteria (for example, *Allochromatium vinosum*) (Figure S2B in Additional file 1). Purple sulfur bacteria are often found in the same lakes where phototrophic consortia occur [31], and the central bacterium may have acquired genes from such organisms. Although sequence analysis cannot determine the attractant or repellent molecule(s) sensed by the products of these genes, purple sulfur bacteria are often positively chemotactic to sulfide [32,33]. The horizontal acquisition of genes for sulfide chemotaxis from a sulfide-oxidizing bacterium could explain how *“Chlorochromatium aggregatum”* gained its known ability to sense and swim towards sulfide [13].

Compared to the genome average, EP_GI-2 has extremely low G + C mol% and includes two genes encoding transposases or integrases, which suggests that these genes were laterally acquired from another organism. Reflecting probable gene transfer between the two partners, five genes in this cluster have very high sequence identities with homologs in the genome of “*Ca.* S. mobilis”, three of which are found in CB_GI-1 (Figure S2A in Additional file 1). Because of the very low G + C mol%, these genes are probably not natively found in either *Chl. chlorochromatii* or “*Ca.* S. mobilis”, and they may have been acquired horizontally by one of the partners and subsequently transferred to the other. Most of the genes in EP_GI-2 and CB_GI-1 are probably involved in cell envelope biosynthesis. These results suggest that these two GIs potentially contain genes essential for establishing close cell-to-cell contact and possibly involved in synthesizing symbiosis-specific structures within the consortium.

### Metabolism and metabolic coupling

**Figure 4:**
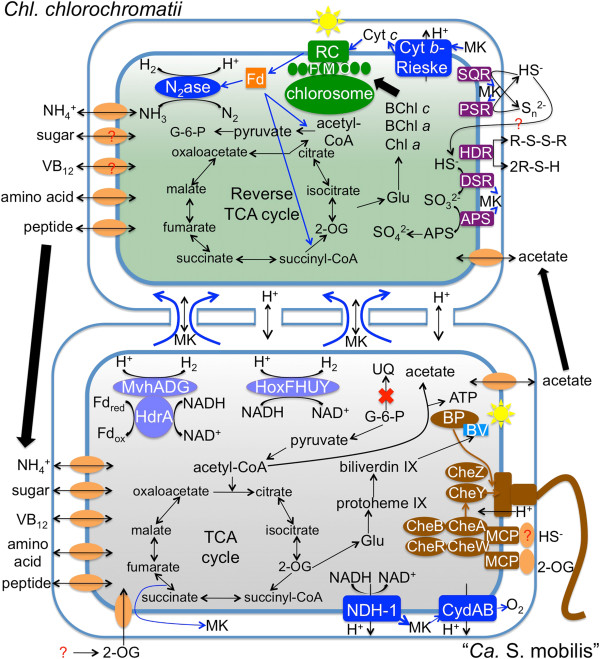
**Cellular overview of central metabolism of *Chl. chlorochromatii *and “*Ca*. S. mobilis”.** Only selected pathways and functions are shown to focus on metabolic coupling, chemotaxis and phototaxis. Blue arrows mark the flow of electrons. Question marks denote unidentified proteins or protein complexes. Abbreviations: 2-OG, 2-oxoglutarate; APS, adenosine 5′-phosphosulfate reductase; Bph, bacteriophytochrome; BV, biliverdin; cyt, cytochrome; DSR, dissimilatory sulfite reductase; FMO, Fenna-Matthews-Olson protein; HDR, heterodisulfide reductase; MK, menaquinone; NDH-1, type 1 NADH dehydrogenase; PSR, polysulfide reductase; Q, quinone; RC, photosynthetic reaction center; SQR, sulfide quinone reductase; UQ, ubiquinone; VB_12_, vitamin B_12_.

Genome analyses suggest that “*Ca.* S. mobilis” has limited metabolic capabilities. Firstly, “*Ca.* S. mobilis” has no pathways for autotrophic CO_2_ fixation, and thus it is a heterotroph that relies on exogenous carbon sources. Secondly, it has very limited pathways for energy production. It lacks recognizable genes for anaerobic respiration with nitrate or sulfate as electron acceptors or for oxidation of inorganic electron donors, except for sulfide:quinone oxidoreductase (SqrA; Cenrod_0552). Thus, “*Ca.* S. mobilis” presumably depends on aerobic respiration or fermentation to produce ATP (but see discussion of interspecies electron transfer below). Compared to its free-living close relatives, “*Ca.* S. mobilis” apparently lost genes for electron transfer proteins such as cytochrome *c*:ubiquinol oxidoreductase, cytochrome *c* oxidase, and most soluble electron carriers (Table S2 in Additional file 2). However, it has retained a complete set of genes for type-1 NADH dehydrogenase and succinate dehydrogenase (Figure 4). The “*Ca.* S. mobilis” genome includes a single terminal oxidase, a cytochrome *bd*-type quinol oxidase, which might allow respiration to occur under the very low O_2_ concentrations (approximately 2.9 μM) found *in situ*[34].

The pairing of an oxygen-sensitive, strict anaerobe and a microaerophile that requires oxygen for some functions is highly unusual. Like other GSB, the epibiont has genes for enzymes involved in protection from reactive oxygen species [35]. Enzymes to protect the cytoplasm of “*Ca.* S. mobilis” from reactive oxygen species include catalase (Cenrod_0449), Fe-Mn superoxide dismutase (Cenrod_1509), alkyl hydroperoxide reductase (Cenrod_0223), and peroxiredoxin (Cenrod_0224, Cenrod_0777, and Cenrod_2189) [35,36]. The expression of some or all of these oxidative stress proteins could be under the control of an OxyR-like, LysR-family transcription factor (Cenrod_2620).

Only two *c*-type cytochromes are encoded by the “*Ca.* S. mobilis” genome. One of these is a small, soluble cytochrome *c*_551_/*c*_552_ (Cenrod_0340), and the other is a periplasmic, diheme cytochrome *c* peroxidase (Cenrod_1795). Although the latter might play a role in protecting the periplasm and cells from the toxic effects of hydrogen peroxide, recent studies in *Shewanella oneidensis* suggest that hydrogen peroxide can also serve as an alternative terminal electron acceptor for dissimilatory energy production [36,37]. In *S. oneidensis*, the electrons for reduction of hydrogen peroxide to water are derived from the quinone pool [37]. In “*Ca.* S. mobilis” a membrane-associated cytochrome *b* (Cenrod_1223) might deliver electrons from menaquinol to cytochrome *c*_551_/*c*_552_, which would serve as the reductant for hydrogen peroxide catalyzed by cytochrome *c* peroxidase.

The “*Ca.* S. mobilis” genome encodes genes for the enzymes of glycolysis, the TCA cycle, and the oxidative pentose phosphate pathway (Figure 4). The absence of lactate dehydrogenase, pyruvate decarboxylase, and pyruvate-formate lyase limits fermentation possibilities involving pyruvate. However, the presence of pyruvate dehydrogenase (Cenrod_2157 and Cenrod_2158), pyruvate:ferredoxin oxidoreductase (Cenrod_0415, Cenrod_0416, and Cenrod_0417), phosphate acetyltransferase (Cenrod_0908), and acetate kinase (Cenrod_0907) suggests that “*Ca.* S. mobilis” can extend the glycolytic pathway beyond pyruvate to acetate, while producing additional ATP by substrate-level phosphorylation. As noted above, under microoxic conditions, respiration could occur by transferring electrons from NADH or menaquinol to oxygen or hydrogen peroxide. The resulting proton-motive force could also be used for ATP synthesis by the F_0_F_1_-type ATP synthase (Cenrod_1756 to 1763).

Under strictly anoxic conditions, protons might be the only available electron acceptor other than CO_2_ (however, see discussion of interspecies electron transfer below). “*Ca.* S. mobilis” encodes two different hydrogenases: a bi-directional group 3d NiFe hydrogenase (Cenrod_0973 and Cenrod_0974) with an associated diaphorase complex (Cenrod_0975 and Cenrod_0976) and a group 3c Mvh hydrogenase (Cenrod_2144, Cenrod_2145, and Cenrod_2148) with an associated heterodisulfide reductase (Cenrod_2147). The diaphorase moiety of the group 3d NiFe hydrogenase should enable the reversible coupling of proton reduction with NADH oxidation [38,39]. During fermentative metabolism, this enzyme could function to reoxidize NADH and reduce protons, but it could alternatively serve as an uptake hydrogenase to oxidize H_2_ produced by the epibionts when they are fixing nitrogen (no uptake hydrogenase is present in the epibiont genome). In methanogens, the Mvh hydrogenase (MvhADG)-heterodisulfide reductase (HdrABC) complex is proposed to couple the exergonic reduction of heterodisulfide CoM-S-S-CoB to coenzyme M (CoM) and coenzyme B (CoB) with the energonic reduction of ferredoxin through H_2_-based electron bifurcation [40-42]. However, the “*Ca.* S. mobilis” genome does not encode homologs of HdrB and HdrC, which form the site of disulfide reduction. This feature, coupled with the absence of evidence for the utilization of CoM and CoB by this taxon, suggests that this enzyme complex has another function. HdrA binds FAD and is thought to be the site of ferredoxin binding. An intriguing possibility is that this enzyme couples the oxidation of NADH (approximately -280 mV) and ferredoxin oxidation (approximately -500 mV) to the reduction of protons. Supporting this possibility, this HdrA subunit (Cenrod_2147) has a NADH binding domain that is not observed in the HdrA subunits of heterodisulfide reductases of methanogens. This might ensure that oxidized pyridine nucleotides are available even when other terminal electron acceptors are not.

*Chl. chlorochromatii* fixes CO_2_ by the reverse TCA cycle, has a complete set of *nif* genes, and can thus fix N_2_ (Figure 4). It excretes large amounts of sugars (mainly glucose) and amino acids (mainly glutamate and aspartate) into the growth medium when grown axenically [43]. In contrast, “*Ca.* S. mobilis” can neither fix N_2_ nor assimilate nitrate or nitrite, but it has an ammonia permease (Cenrod_1218) and several sugar and amino acid transporters (Figure 4). It therefore seems likely that sugars and amino acids are transferred from the photoautotrophic epibionts to heterotrophic “*Ca.* S. mobilis”.

2-Oxoglutarate stimulates the growth of *“Chlorochromatium aggregatum”*[13], and another phototrophic consortium, *“Pelochromatium roseum”*, incorporated 2-oxoglutarate *in situ*[44]. However, 2-oxoglutarate had no effect on the growth of *Chl. chlorochromatii*[14], suggesting that only “*Ca.* S. mobilis” assimilates 2-oxoglutarate. Consistent with this idea, the “*Ca.* S. mobilis” genome encodes one TRAP-type dicarboxylate transporter (Cenrod_1182, Cenrod_2378 and Cenrod_2379). Growth of *Chl. chlorochromatii* is stimulated by photo-assimilation of acetate [14], which is probably produced from pyruvate by oxidation of sugars or 2-oxoglutarate by “*Ca.* S. mobilis” (see above). These types of metabolite exchange would be mutually beneficial.

“*Ca.* S. mobilis” probably takes up amino acids released by *Chl. chlorochromatii*, and two ABC transporters for ‘branched-chain’ amino acids (Cenrod_0106 to 0109; Cenrod_2184, Cenrod_2264, Cenrod_2266, and Cenrod_2267), as well as other amino acid transporters, are encoded in its genome. Nevertheless, it has not generally abandoned its ability to synthesize amino acids but has streamlined some pathways (Figure 3). For example, “*Ca.* S. mobilis” cannot perform assimilatory sulfate reduction, but can synthesize cysteine and methionine from sulfide. Instead of using two enzymes, a single protein (Cenrod_2045) similar to aminotransferases AspC and TyrB probably performs both activities. 3-Phosphoglycerate dehydrogenase (SerA) appears to be missing, but it is probably premature to conclude that “*Ca.* S. mobilis” cannot synthesize serine [45]. “*Ca.* S. mobilis” also probably depends on the epibiont for essential cofactors. “*Ca.* S. mobilis” has a heterodimeric, MetH-type, cobalamin-dependent methionine synthase (Cenrod_2368 and Cenrod_2596) but lacks the genes for cobalamin synthesis. However, it has a putative cobalamin transport system, and this suggests that it obtains vitamin B_12_ from the epibiont, which has all genes required for cobalamin synthesis. “*Ca.* S. mobilis” apparently obtains menaquinone from *Chl. chlorochromatii* as well (see below). In summary, as in many other symbiotic systems [46], metabolic dependence - mainly of “*Ca.* S. mobilis” on *Chl. chlorochromatii -* is apparently an important component of the relationship between these two partners (Figure 4).

### Chemotaxis, phototaxis, and signal transduction

Metabolic coupling is obviously critical for the survival of “*Ca.* S. mobilis”; however, *Chl. chlorochromatii* is a photolithoautotroph and does not appear to gain much from such coupling. On the other hand, the motility of the consortium provides a huge advantage to the epibiont over free-living relatives. Swimming motility has not been reported for any GSB, and planktonic GSB with gas vesicles can only reposition themselves slowly [47]. Flagella-powered taxis towards sulfide and away from darkness towards light - which are the main energy and electron sources of GSB - would allow consortia to adjust more quickly to fluctuating light and oxygen conditions during the diel cycle in their natural habitats [34]. Diel vertical migration behavior is highly advantageous for the flagellated purple sulfur bacterium *Chromatium minus*[48], and directed motility is generally regarded as one of the major advantages that allow phototrophic consortia to outcompete free-living GSB [49].

**Figure 5:**
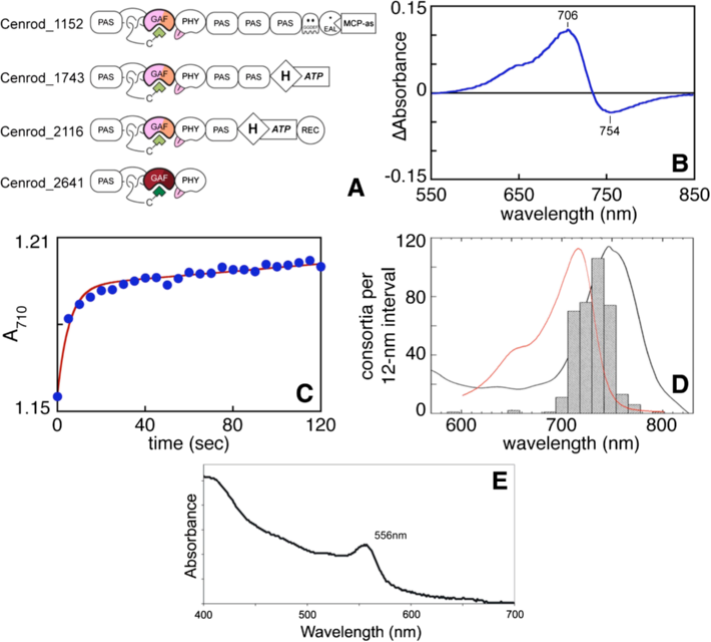
**Photosensors of “*Ca. *S. mobilis”. (A)** Domain structures of the four bacteriophytochromes encoded in the genome of “*Ca.* S. mobilis”. The experimentally verified red/far-red photocycle and BV chromophore of Cenrod_2641 are indicated by the darker colors; hypothetical photocycles are in faded colors. **(B)** Red/far-red photocycle of Cenrod_2641. The gene corresponding to Cenrod_2641 was co-expressed with heme oxygenase, which produces biliverdin (BV) from heme when expressed in *E. coli*. Purified Cenrod_2641 was characterized by absorbance spectroscopy before and after illumination with 700 ± 20 nm light. The (Before - After) difference spectrum is shown, with peak wavelengths indicated. **(C)** Dark reversion of the 750 nm photoproduct was characterized. Cenrod_2641 was held at photoequilibrium under 700 nm illumination. Illumination was then discontinued, and absorbance at 710 nm was monitored as a function of time. Data were biphasic, with the second phase only poorly resolved due to low signal-to-noise. We therefore fit the data to a single exponential with a linear second phase (equivalent to burst kinetics), deriving a rate constant of 0.2 s^-1^ for the fast initial phase. **(D)** Comparison of the dark-adapted absorption spectrum of Cenrod_2641 (red curve, arbitrary scale) to the absorption spectrum of intact consortia (black curve, arbitrary scale) and to the integrated number of accumulated consortia per 12 nm interval (bars, the scotophobotactic response of the consortia) (data from [13]). **(E)**. Absorption spectrum of recombinant CpcA-PEB produced in *Escherichia coli*. BV, the precursor of phycoerythrobilin (PEB), was produced by Cenrod_2641 (heme oxygenase) and converted to PEB when cells were grown under oxic conditions. No red-colored/gold-fluorescent CpcA-PEB was produced under anoxic conditions, showing that the heme oxygenase encoded by Cenrod_2642 requires oxygen as a co-substrate for heme cleavage.

Microscopic analyses have shown that “*Ca.* S. mobilis” cells possess a single polar flagellum that confers motility to the consortia [34]. Because two of the strongest attractants, light and sulfide, provide no apparent direct benefit to “*Ca.* S. mobilis”, it had been proposed that these attractants were sensed by *Chl. chlorochromatii*, and that a signal was then transmitted to “*Ca.* S. mobilis” [13]. The genomic data suggest instead that the genome of the central rod contains the dedicated sensory proteins of the consortium, with a complex photosensory apparatus having similarity to those of cyanobacteria and purple photosynthetic bacteria. In the cyanobacterium *Synechocystis* sp. PCC 6803, multiple photoreceptors have been implicated in regulating phototaxis, with the domain structure of these sensors implicating multiple signaling pathways. PixD is a member of the blue-light-sensor-using-flavin (BLUF) family with no obvious signaling domains, but it is nevertheless able to interact with a response regulator (REC) protein, PixE [50]. Three members of the phytochrome superfamily are also known to function in determining *Synechocystis* sp. PCC 6803’s responses to blue light [51-55]. One of these proteins, SyPixJ, possesses a carboxy-terminal methyl-accepting-chemotaxis-protein (MCP in Figure 5) domain that is also found in chemotactic signaling proteins. Another, UirS or PixA, has a bipartite histidine kinase domain (H/ATP), while the third protein, Cph2, can function as a sensor for both red and blue light and contains GGDEF and EAL domains associated with metabolism of the bacterial second messenger molecule cyclic-di-GMP. Reported phototactic responses to red light in *Synechocystis* sp. PCC 6803 [56] have not been unambiguously assigned to a given photosensor, but this organism possesses additional histidine kinases in the phytochrome superfamily that exhibit the requisite spectral response [57,58]. Bacteriophytochrome members of this superfamily are also responsible for responses to red and far-red light in the anoxygenic photosynthetic bacterium *Rhodopseudomonas palustris*[59].

Members of the phytochrome superfamily are thus likely candidate sensors for red-light phototaxis in consortia. Such proteins require linear tetrapyrrole (bilin) chromophores synthesized from heme for photosensory function; in particular, bacteriophytochromes incorporate biliverdin (BV) [60-62]. BV is synthesized from heme by heme oxygenase [63]. Although *Chl. chlorochromatii* completely lacks genes encoding known photosensors, the “*Ca.* S. mobilis” genome encodes four bacteriophytochromes (Cenrod_1152, Cenrod_1743, Cenrod_2116, and Cenrod_2641) and heme oxygenase (Cenrod_2642) (Figure 5A). The bacteriophytochrome genes encode the conserved PAS-GAF-PHY photosensory region required for photoperception by phytochromes, but they exhibit diverse domain architectures that implicate signaling pathways similar to those reported for *Synechocystis* sp. PCC 6803 (Figure 5A). There are two histidine kinases, one of which also contains a REC domain. Another bacteriophytochrome has GGDEF, EAL, and MCP domains. The fourth protein, Cenrod_2641, lacks apparent output domains, like PixD from *Synechocystis* sp. PCC 6803. Cenrod_2641 is encoded in an apparent operon with the sole heme oxygenase gene (Cenrod_2642) found in the “*Ca.* S. mobilis” genome. The “*Ca.* S. mobilis” genome thus strongly implicates bacteriophytochromes encoded by the central rod as sensors for phototaxis, with multiple signaling pathways responding to red/far-red light. We expanded on these results by confirming that one of the putative photosensory proteins (Cenrod_2641) encoded by the “*Ca.* S. mobilis” genome, as well as its cognate heme oxygenase (Cenrod_2642), are functional.

We heterologously expressed Cenrod_2641 in *Escherichia coli* strains that allowed the co-expression of BV or two other linear tetrapyrroles, phycocyanobilin and phytochromobilin [64]. Recombinant bacteriophytochromes produced in these three strains were purified and characterized by absorbance spectroscopy. All three samples had similar absorption spectra with maximal absorption at approximately 710 nm, which suggested that only biliverdin was efficiently incorporated into the protein [65]. Similar to other phytochromes [66,67], Cenrod_2641 photoswitched between a thermally stable dark state and a photoproduct having distinct spectral properties. Upon illumination of the long-wavelength absorption band with 700 nm light, peak absorbance at 706 nm decreased with concomitant formation of a photoproduct at 754 nm (Figure 5B). The photoproduct rapidly converted back to the dark form in the dark (Figure 5C); this process was sufficiently rapid to allow Cenrod_2641 to function as an effective intensity sensor for far-red light [58,68,69]. These results showed that Cenrod_2641 has photochemical properties compatible with those of a *bona fide* photosensor with high affinity for BV.

 Figure S3 in Additional file 1 shows a phylogenetic tree that includes bacteriophytochrome sequences from a variety of bacteria, most of which are found in members of the Proteobacteria. The Cenrod_2641 bacteriophytochrome produces a clade with sequences from two members of the genus *Methylomicrobium*. Although distantly homologous bacteriophytochromes occur in some members of the Comamonadaceae as well as other Betaproteobacteria, the bacteriophytochrome encoded by Cenrod_2641 is nested among sequences from Gammaproteobacteria. This result suggests that the gene encoding this bacteriophytochrome (and probably the associated heme oxygenase; data not shown) were acquired by “*Ca.* S. mobilis” via horizontal gene transfer from a member of the Gammaproteobacteria.

The putative heme oxygenase (Cenrod_2642) was substituted for a functional cyanobacterial heme oxygenase in an *E. coli* strain that can produce a highly fluorescent CpcA protein when BV is converted into phycoerythrobilin (PEB) by PebS and ligated to CpcA by the phycobiliprotein lyase, CpcE/CpcF [64]. As shown in Figure 5E, PEB was produced and ligated to CpcA (λ_max_ = approximately 556 nm) when Cenrod_2642 functionally replaced heme oxygenase in this system under oxic conditions but not under anoxic conditions (data not shown). These results confirm that “*Ca.* S. mobilis” produces BV for one and up to four bacteriophytochromes, which should allow photoperception in the wavelength range 700 to 760 nm. This wavelength range matches the action spectrum for the scotophobic response of *“Chlorochromatium aggregatum”* (Figure 5D) [13].

Although light sensing by non-phototrophic bacteria has previously been described in several organisms, in most cases it is associated with protection from reactive oxygen species produced by the interaction of light and photosensitizing molecules [60,70]. The occurrence of scotophobotaxis by a non-phototrophic bacterium - that is, taxis to *remain* in the light - is extremely rare if not unprecedented. This finding does not completely exclude the original proposal that light is absorbed by chlorosomes and other components of the photosynthetic apparatus of the epibiont cells, which then transmit a signal to “*Ca.* S. mobilis”. The scotophobic response in “*Ca.* S. mobilis” may have initially been selected and fine-tuned by metabolic signaling (for example, metabolite transfer) from the epibiont to the central rod in the consortium (see below). However, light sensing mediated by bacteriophytochrome(s) is obviously expected to be more direct and to have a more rapid effect on the swimming behavior of “*Ca.* S. mobilis”.

“*Ca.* S. mobilis” also appears to sense many chemical signals. The “*Ca.* S. mobilis” genome is enriched in chemotaxis genes, and most of these genes occur in multigene families, including 33 MCP proteins. It is difficult to predict attractants or repellents for non-photosensory MCPs. Some MCP genes are clustered with genes encoding periplasmic solute-binding proteins, which suggests that these MCPs function as signal transducers. Proteins putatively binding phosphate (Cenrod_1484), amino acids (Cenrod_0096) and oligopeptides (Cenrod_0130) were clustered with MCPs, which strongly suggests that cells can probably sense and swim towards (or away from) these compounds. A binding protein for dicarboxylates (Cenrod_1184) might also interact with MCPs, and this might explain chemotaxis of *“Chlorochromatium aggregatum”* towards 2-oxoglutarate. It is unknown whether chemotaxis towards sulfide is achieved through a similar mechanism, because proteins for sensing sulfide have not yet been identified. However, given the current lack of understanding about such proteins, sulfide sensing remains a strong possibility for these proteins. Chemotaxis towards compounds excreted by the epibiont, for example, amino acids, could indirectly contribute to taxis towards sulfide, because the excretion of amino acids and other fixed carbon and nitrogen compounds by the epibiont should directly depend on the availability of light and sulfide. However, responses to excreted compounds would probably be much slower than any intracellular mechanism(s) possessed by “*Ca.* S. mobilis”.

Chemical signals sensed by the binding proteins could also have effects on cellular processes other than taxis. Signal transduction between “*Ca.* S. mobilis” and *Chl. chlorochromatii* is suggested by at least one previous experiment [44]. Epibiont and central rod cells divide synchronously, and this is presumably coordinated by signaling molecules exchanged between the partners. Such signal transduction might be achieved by mechanisms similar to that of chemotaxis. It is possible that some of the above-mentioned MCP-like proteins participate in regulation of cell cycles as in *Myxococcus xanthus*[71]. In addition to MCPs, many genes encoding membrane-bound signal transduction proteins, such as two-component system proteins or proteins containing EAL and/or GGDEF domains, are often clustered with periplasmic solute-binding proteins. Proteins containing these domains have been shown to participate in swarming and cell surface adhesion [72], which is essential for formation of consortia.

### Potential interspecies electron transfer

Besides metabolite exchange and motility, interspecies electron transfer would be extremely advantageous to the consortium in its energy-limited niche. *Chl. chlorochromatii* relies on having a constant supply of sulfide to provide the electrons it requires for carbon dioxide fixation and growth. Any interaction that increases the availability of sulfide (or electrons) would enhance growth. It was once thought that a sulfur cycle might occur between the two partners. The central bacterium might reduce oxidized sulfur compounds and return the products to the epibiont [73], which would be similar to the sulfur cycling that occurs in a syntrophic co-culture of *Chlorobium vibrioforme* and *Desulfuromonas acetoxidans*[74]. This hypothesis was supported by an *in situ* study that concluded that the increase in biomass by *“Pelochromatium roseum”* far exceeded the maximum possible CO_2_ fixation that could be associated with oxidation of sulfide reaching the chemocline from below [34]. Direct photo-assimilation of acetate and electrons derived from sulfur cycling between consortia and associated sulfate-reducers were suggested as mechanisms to overcome this shortfall. However, because “*Ca.* S. mobilis” is a member of Betaproteobacteria, and within the Proteobacteria all known sulfate-reducers are members of the Deltaproteobacteria, a sulfur cycling mechanism seemed unlikely to occur in the consortia*.* The genomic data are consistent with predictions based on phylogenetic associations. The “*Ca.* S. mobilis” genome does not contain known genes for the reduction of sulfate or sulfur to sulfide [75].

The genomic data were searched for other possible mechanisms of interspecies electron transfer. However, other than the two NiFe-hydrogenases described above, only one other set of electron transfer-related proteins was identified. The “*Ca.* S. mobilis” genome encodes six genes (Cenrod_1907 to 1912) that have some sequence similarity to subunits of formate hydrogenlyase (Hyf subunits B, C, E, F, G, and I) [76] as well as modules of some other electron transfer complexes, including the Mbx complexes thought to be involved in sulfur reduction [77]. The occurrence of highly similar gene clusters in more than 300 bacteria, including free-living members of the *Comamonadaceae* that do not live in sulfidic environments, strongly suggests that this complex is not involved in sulfur reduction. Cenrod_1908 exhibits homology with the large subunit of NiFe-hydrogenase but lacks the required L1 and L2 cysteine ligands required to coordinate the NiFe cofactor [78], which implies that this complex is not involved in H_2_ cycling. This complex possibly plays a role in coupling menaquinone oxidation/reduction to proton translocation, but the redox partner for this process and its directionality are presently uncertain.

**Figure 6:**
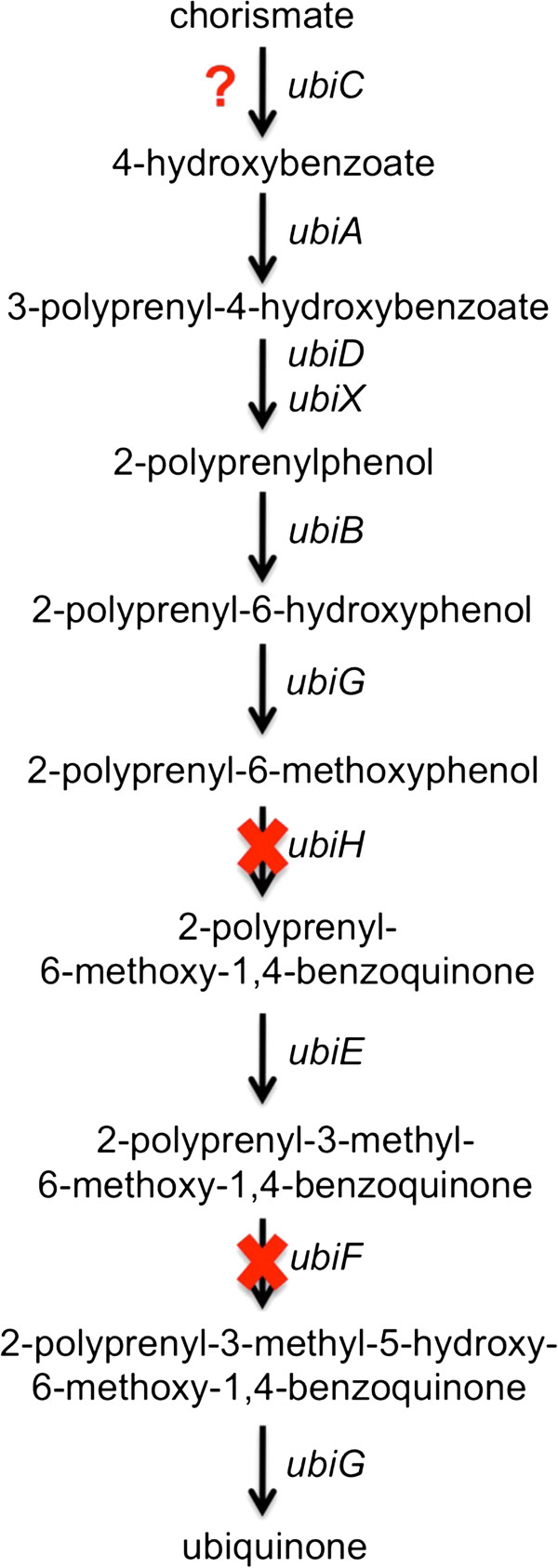
**Ubiquinone biosynthesis pathway of “*Ca*. S. mobilis”.** This pathway is compared with the ubiquinone biosynthesis pathways found in 15 members of the *Comamonadaceae,* including the 8 listed in the Figure 2 legend as well as 3 additional *Acidovorax* spp., 2 additional *Comamonas* spp., one additional *Polaromonas* sp. and one additional *Variovorax* sp. The *ubiC* gene was identified in only one of the 15 genomes, possibly because of its extremely low sequence similarity and conservation across species. The *ubiH/coq6* and *ubiF/coq7* genes are highly conserved and were identified in each of the 15 reference genomes, but these genes were not found in “*Ca*. S. mobilis”.

Quinone exchange is a potential mechanism for electron shuttling between the two partners of the consortium. “*Ca.* S. mobilis” lacks genes for either of the two known pathways for menaquinone biosynthesis [79-81], and it has an incomplete ubiquinone biosynthesis pathway. However, all free-living members of the *Comamonadaceae* can synthesize ubiquinone, and these organisms universally have two genes, *ubiH/coq6* and *ubiF*/*coq7*, which encode oxygen-dependent enzymes that are missing from the genome of “*Ca.* S. mobilis” (Figure 6; Table S2 in Additional file 2). Either “*Ca.* S. mobilis” lacks the ability to synthesize isoprenoid quinones, or it has acquired or evolved unknown genes to replace the activities of these two missing enzymes. The latter seems highly unlikely because only menaquinone-7 and no ubiquinone was detected in intact consortia and cell fractions enriched in “*Ca.* S. mobilis” (Figure S3 in Additional file 1).

The genomic data, however, strongly suggest that “*Ca.* S. mobilis” cells utilize quinones. At least four important electron-transport complexes (type-1 NADH dehydrogenase, succinate dehydrogenase, cytochrome *bd*-type quinol oxidase, and sulfide:quinone reductase) that are encoded in the “*Ca.* S. mobilis” genome require a quinone substrate. The presence of these genes strongly suggests that quinones are available to the central bacterium; otherwise, these genes should have been partially or completely lost. Extracellular transfer of quinones has previously been described from wild-type *Shewanella putrefaciens* to a mutant that was unable to synthesize menaquinone [82]. Similarly, respiration can be activated in Group B *Streptococcus* spp. by menaquinone synthesized by *Lactococcus lactis*[83]. Finally, exchange of a water-soluble intermediate, 1,4-dihydroxy-2-naphthoic acid, can satisfy the quinone requirement of members of dental plaque [84]. Thus, we hypothesize that menaquinone-7, or a soluble quinone intermediate synthesized by *Chl. chlorochromatii*, can be transferred to “*Ca.* S. mobilis”.

The availability of quinones for respiration in “*Ca.* S. mobilis” solves only one of two problems. Consortia are usually cultivated under strictly anoxic conditions in the laboratory and thus should not have any terminal electron acceptor for respiration. Although “*Ca*. S. mobilis” can probably produce ATP by fermentation of glucose under anoxic conditions as described above, an energetically more favorable solution would involve bidirectional quinone transfer and sharing of the resulting protonmotive force. Quinones transferred to “*Ca*. S. mobilis” would allow energetically more favorable electron transfer processes to occur in the central bacterium, and electrons returned by quinols to the epibiont could be reused for CO_2_ fixation. Although this process does not involve sulfide, it produces results similar to sulfur cycling. The exchange of electrons and shared proton-motive force would be beneficial to both symbiotic partners and would additionally allow ATP synthesis in the central rod to be directly coupled to the light reactions of photosynthesis in the epibiont. This might partly explain the phototactic and chemotactic behavior of the central bacterium, and this could additionally explain the substantial loss of genes for energy metabolism from the central bacterium (Figure 3). Fermentation could still provide ATP for the central bacterium under anoxic conditions in the dark.

For either sulfide- or quinone-dependent electron shuttling and shared proton-motive force to occur, symbiosis-specific, specialized cell wall structures would likely be required. The central rod and epibiont cells are joined by numerous ‘periplasmic tubules’ [17], which are reminiscent of the bacterial nanowires that have recently been found in many organisms, including *Geobacter sulfurreducens* and *Shewanella oneidensis* and between organisms in syntrophic co-cultures of *Pelotomaculum thermopropionicum* and *Methanothermobacter thermoautotropicus*. Electrically conductive nanowires in these organisms have been proposed to be conduits for extracellular or interspecies electron transfer [85,86]. The electron carriers in some cases appear to be cytochromes, but other electron carrier molecules could function in organisms devoid of cytochromes [85,87]. The periplasmic tubules of phototrophic consortia could have similar functions, and quinone-like molecules, whether soluble or shuttled by proteins, might facilitate electron exchange between “*Ca.* S. mobilis” and *Chl. chlorochromatii*. Periplasmic tubules are larger in diameter than pili and bacterial nanowires, and they connect the outer membranes of the two cell types, thus creating a joint periplasmic space between the epibionts and central bacterium [17]. Periplasmic tubules thus provide a mechanism for sharing proton-motive force generated by the proposed electron shuttle between the two organisms. The potential schemes for interspecies electron transfer hypothesized here will obviously have to be tested experimentally in the future. Nevertheless, they provide an additional perspective for the symbiotic relationship of phototrophic consortia. Electron shuttling and shared protonmotive force would join metabolite exchange and phototaxis/chemotaxis in creating a strong, competitive advantage for consortia over free-living members of the GSB.

## Conclusions

Genomic data for the phototrophic consortium *“Chlorochromatium aggregatum”* suggest that a very sophisticated symbiotic relationship has evolved between the central bacterium, “*Ca.* S. mobilis”, which apparently is no longer capable of independent growth, and the epibiont, *Chl. chlorochromatii*, which is still capable of independent growth. We propose that three types of interactions occur between the two partners (Figure 4). Firstly, metabolite exchange, which is common in many other symbiotic organisms, also occurs in this consortium, but the wide variety of exchanged metabolites, including carbon, nitrogen and sulfur sources and vitamins, is uncommon [40]. Secondly and remarkably, “*Ca.* S. mobilis” can sense light and probably sulfide, which are most directly beneficial to *Chl. chlorochromatii*. “*Ca.* S. mobilis” can also sense other nutrients and probably the metabolic status of *Chl. chlorochromatii*. Figuratively, *Chl. chlorochromatii* cells are the solar panels of this self-perpetuating, solar-energy-powered bacterial machine; “*Ca.* S. mobilis” not only provides the bus but also the driver and a navigation system. The degree of specialization observed for these two organisms approaches that seen in multicellular organisms. Although phototrophic consortia are composed of two different organisms, studies of these consortia might offer insights into the evolutionary processes that led from single-celled to multicellular organisms. Thirdly, electron cycling mechanisms, particularly those mediated by quinones and potentially shared proton-motive force, could provide important new mechanistic bases for energy exchange in symbiotic relationships. This study provides many novel insights for this specific bacterial symbiosis, but it also reveals benchmarks for understanding other phototrophic consortia, bacterial symbioses in general, and more complex communities and multicellularity.

## Materials and methods

### Culture and DNA preparation

Genomic DNA of the epibiont was extracted from an axenic culture of *Chl. chlorochromatii* strain CaD3 [14]. The central bacterium has not yet been grown axenically. Consortia were grown under conditions that produced a biofilm on a glass surface, which could be recovered and which reduced contamination from other bacteria in the enrichment culture. The consortia cultures were pelleted and resuspended in K4 medium lacking H_2_S and NaHCO_3_ to an OD_650nm_ = 4. To disaggregate the biofilm and consortia into single cells, a cell suspension was incubated in a water bath at 68°C for 10 minutes. During the incubation, aggregates were disrupted by passing the culture through a syringe (0.80 mm × 120 mm) several times. Subsequently, the central bacterial cells were separated from the epibiont by CsCl equilibrium density centrifugation [18]. The two distinct bands containing cells of the epibiont and central bacterium were removed with a syringe and needle (0.80 mm × 120 mm) and aliquots (20 μl) of cells were removed from each fraction for fluorescent *in situ* hybridization (FISH) analysis to assess the level of cross-contamination in each fraction [18]. The remaining volume was mixed with sterile double-distilled water, pelleted and rapidly frozen in liquid nitrogen. A fraction highly enriched in “*Ca.* S. mobilis” was used to extract DNA used for genome sequencing of “*Ca.* S. mobilis”.

### Sequencing and assembly

The genome of *Chl. chlorochromatii* was sequenced using the whole genome shotgun sequencing approach and the assembly and finishing process have been described elsewhere [16]. The enriched genomic DNA sample from “*Ca.* S. mobilis” was sequenced using pyrosequencing technology (GS-20 FLX; 454 Life Sciences, Branford, CT, USA). A total of 1,064,718 sequences, averaging 362 bp per sequence, were generated. Sequences with at least 98% nucleotide sequence identity to *Chl. chlorochromatii*, defined by alignment to its genome, were removed before the remaining sequences were assembled using the Newbler program (454 Life Sciences). About 23% of the total sequences were removed in this filtering process. A different approach, in which *Chl. chlorochromatii* contigs were removed after all sequences had been assembled, resulted in very similar assemblies. The average read depths of the former assembly, which contained 3,508 contigs, was examined: 121 contigs had read depths averaging approximately 83×, while the remainder had much lower read depths (approximately 4× to 6× on average). Because genes associated with these 121 contigs appeared to be closely related to those of Betaproteobacteria, these contigs were tentatively assigned to “*Ca*. S. mobilis”. The other contigs probably arose from other organisms in the enrichment culture. These 121 contigs, which were assembled from 668,485 sequences (63% of original sequence pool), were used for subsequent assembly and finishing. The original trace files for sequencing the “*Ca*. S. mobilis” genome have been deposited in the Sequence Read Archive at GenBank under the Biosample accession number SRS500535.

For finishing, a set of sequences that were derived from Sanger sequencing of clone libraries with 3 and 8 kb inserts and produced from total genomic DNA from *“Chlorochromatium aggregatum”* by the DOE Joint Genome Institute were used. A total of 9,798 paired-end sequences with at least 95% nucleotide sequence identity to the 121 initial contigs were extracted and added to the assembly dataset to predict contig arrangements into scaffolds. Based on predictions from these analyses, PCR amplicons were produced and sequenced to close most of the 121 gaps in the scaffolds. PCR amplicons for other gaps were obtained using multiplex PCR, TAIL-PCR or combinatorial PCR [88]. The finishing and polishing process was carried out using the Phred/Phrap/Consed software package [89,90]. The genome of *Chl. chlorochromatii* was annotated by the Joint Genome Institute and deposited in GenBank (accession number CP000108). The genome of “*Ca*. S. mobilis” was annotated using a previously described pipeline [91] and has also been deposited in GenBank (accession number CP004885).

### Identification of genomic islands

The genomic islands were first predicted using the program Alien_hunter [92]. A score cutoff of 25 and a size cutoff of 10 kb were used. Predicted islands were manually inspected to eliminate falsely identified regions such as rRNA and extremely conserved proteins (ribosomal proteins) because of their naturally biased sequence composition. Boundaries of remaining islands were then carefully optimized by inspecting BLASTX hits of genes in the islands in the NCBI nr database. Genes near the ends of islands were removed if they were closely related to proteins of related organisms or were added if the opposite was true.

### Expression and characterization of bacteriophytochrome

A putative bacteriophytochrome gene (Cenrod_2641) was amplified by polymerase chain reaction from DNA enriched from the central rod using primers: CRBP1F (5′-GAGCACCCTCATATGACCGACATGATCCTGATC-3′) and CRBP1R (5′-TGGTTTGAATTCTCATAGCCAGCGTAGAAGGTT-3′). The resulting product was then digested with NdeI and EcoRI and ligated into similarly digested pBS405v [93]. This construct allowed the expression of the putative bacteriophytochrome with a hexa-His tag. The resulting plasmid was cotransformed into *E. coli* BL21 (DE3) with plasmid pHO1, which directs the synthesis of biliverdin from heme [64].

A putative heme oxygenase (Cenrod_2642) was PCR amplified from central rod enriched DNA using primers CRHOF (5′-GCTGCACCGCCATATGCGGCAAGCAAGCAAGTTG-3′) and CRHOR (5′-ACCAGTGATATCTCACTGCCCCGCTGCTGCC-3′), digested with NdeI and EcoRV, and cloned into similarly digested pACYC Duet-1 (Novagen, Madison, WI, USA) to create the plasmid pACYC-CRHO. In order to create a plasmid with both Cenrod_2642 and *pebS*, the plasmid pPebS [93] was digested with BamHI and NotI and the resulting 719 bp fragment containing *pebS* was cloned into similarly digested pACYC-CRHO. This new plasmid, pACYC-CRHOpebS, was then co-transformed along with the plasmid pBS414v [94] into *E. coli* BL21 (DE3) cells. This strain was visually assayed for its ability to produce a functional phycoerythrobilin chromophore that could be attached to CpcA as described previously [64]. The *E. coli* cells were bright red in color when grown under oxic conditions but were uncolored when grown under anoxic conditions. These results showed that the putative heme oxygenase was functional and produced biliverdin when oxygen was present [64].

Absorbance spectroscopy and photochemistry were carried out at 25°C using a Cary 50 spectrophotometer and a 75 W Xe lamp as previously described [95]. Illumination was restricted to 700 ± 20 nm or 650 ± 20 nm with interference band-pass filters (CVI Melles Griot).

### HPLC analyses of quinones

Quinones were extracted from cells with methanol:acetone (7:2, vol:vol), analyzed by reversed-phase high-performance liquid chromatography as previously described [96,97], and detected by absorption at 270 nm. Reference compounds ubiquinone-8 and menaquinone-8 were extracted from *E. coli* cells grown under oxic or anoxic conditions, respectively, and menaquinone-7 was obtained from *Chlorobaculum tepidum* cells [96]. Only menaquinone-7 was detected in extracts prepared from intact consortia and fractions enriched in the central bacterium.

## Abbreviations

bp: base pair; BV: biliverdin; Ca.: *Candidatus*; CB_GI: genomic island in central bacterium “*Ca*. S. mobilis”; Chl.: *Chlorobium*; EP_GI: genomic island in epibiont *Chl. chlorochromatii*; GI: genomic island; GSB: green sulfur bacteria; MCP: methyl-accepting chemotaxis protein; ORF: open reading frame; PEB: phycoerythrobilin; TCA: tricarboxylic acid cycle.

## Competing interests

The authors declare that they have no competing interests.

## Authors’ contributions

SCS, JO and DAB designed research. ZL, JM, TL, RMA, KV, N-UF, NCR, LPT, SCS, PH, and MR performed research. ZL, TL, N-UF, ESB and DAB analyzed data. ZL, N-UF, NCR, JO and DAB wrote the paper. All authors read and approved the final manuscript.

## Supplementary Material

Additional file 1: Figure S1 Scanning electron micrographs (A-E) and epifluorescence (F-H) photomicrographs of ‘*Chlorochromatium aggregatum.*’ **Figure S2**. Genes in selected genomic islands of *Chl. chlorochromatii* and “*Ca*. S. mobilis”. **Figure S3**. Phylogenetic analysis of one bacteriophytochrome gene (Cenrod_2641) and its most similar homologs in the database and those in taxonomically related organisms. **Figure S4**. Quinone analysis of ‘*Chlorochromatium aggregatum*’. Click here for file

Additional file 2: Table S1 Genes unique to *Chl. chlorochromatii* that are not found in other GSB. **Table S2**. Conserved orthologous *Comamonadaceae* genes that are missing in the genome of “*Ca.* S. mobilis”. **Table S3**. Genes unique to “*Ca*. S. mobilis*”* that do not have orthologs in 8 other *Comamonadaceae* genomes. **Table S4**. Potential horizontal transferred gene pairs of “*Ca.* S. mobilis” and *Chl. chlorochromatii*. Click here for file
