# Supplementary material for: Genomic analysis reveals key aspects of prokaryotic symbiosis in the phototrophic consortium “Chlorochromatium aggregatum”
Source: Genome Biol. 2013 Nov 22;14(11):R127. doi: 10.1186/gb-2013-14-11-r127 (PMC4053972; doi:10.1186/gb-2013-14-11-r127)
Supplement: Additional file 1: Figure S1 — Scanning electron micrographs (A-E) and epifluorescence (F-H) photomicrographs of ‘Chlorochromatium aggregatum.’ Figure S2. Genes in selected genomic islands of Chl. chlorochromatii and “Ca. S. mobilis”. Figure S3. Phylogenetic analysis of one bacteriophytochrome gene (Cenrod_2641) and its most similar homologs in the database and those in taxonomically related organisms. Figure S4. Quinone analysis of ‘Chlorochromatium aggregatum’. [file gb-2013-14-11-r127-S1.pdf]

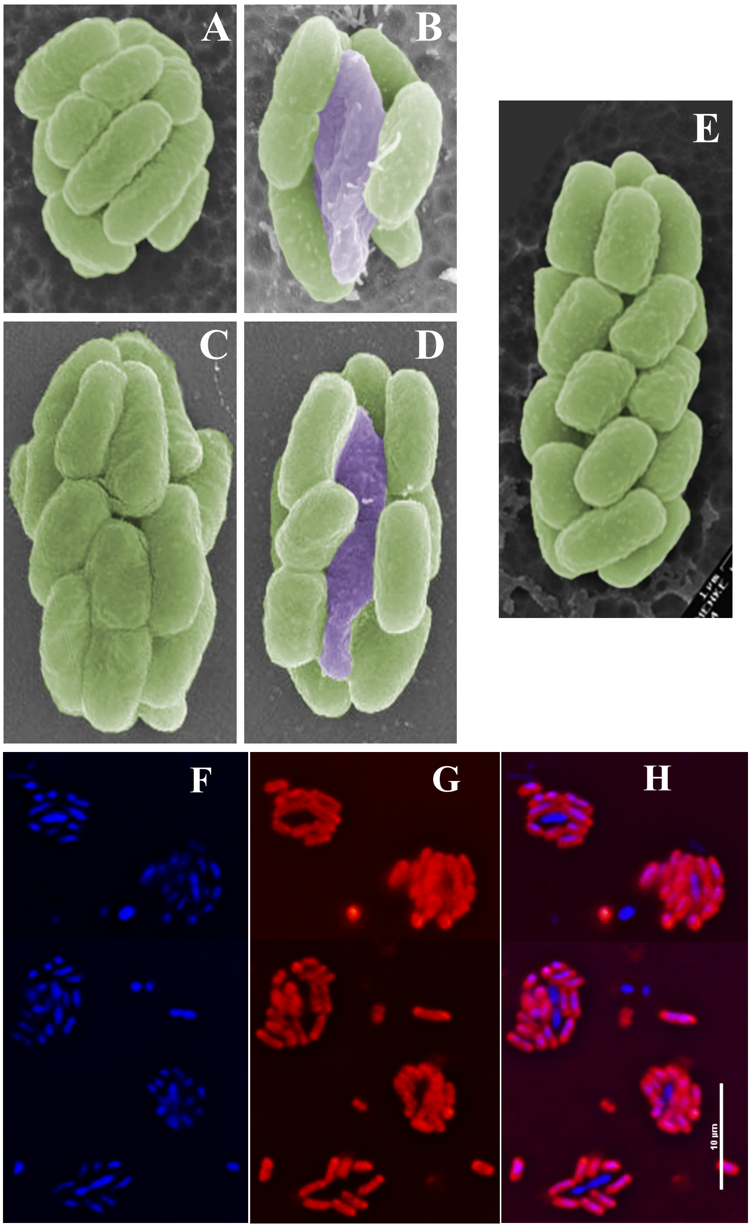

Supplemental Figure S1. Scanning electron micrographs (A-E) and epifluorescence (F-H) photomicrographs of "*C. aggregatum.*" Epibionts are shown in false color green, central bacteria in false color purple in A to E. (A) Consortium in an early stage of cell division, also seen in (B) with a partially uncovered central bacterium. Consortia with elongated epibiont cells (C), and elongated central bacterium (D), and during division into two daugther consortia (E). (F) Micrograph of consortia stained with (DAPI). (G) Micrograph showing the autofluorescence (excitation wavelength, 445 nm; emission wavelength, >740 nm) of bacteriochlorophyll *c* present in the epibiont chlorosomes. (H) Overlay of F and G. The white bar in panel K equals 10 µm.

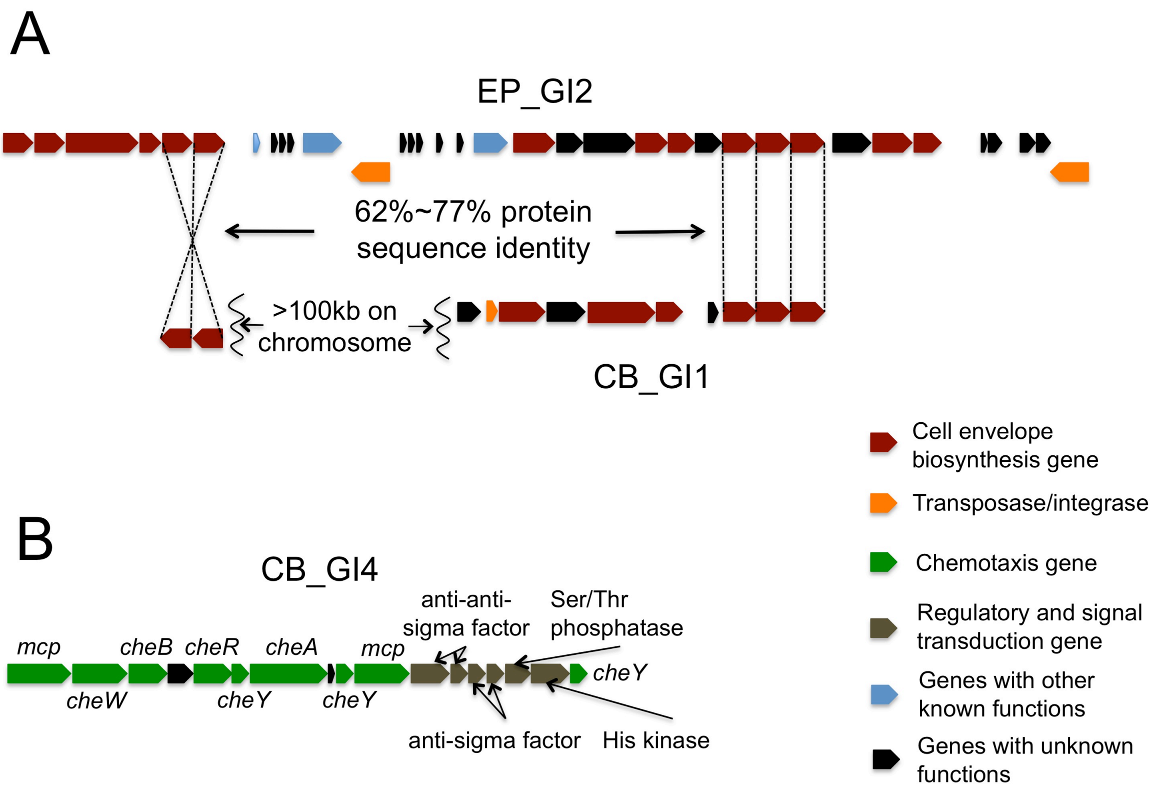

Supplemental Figure S2. Genes in selected genomic islands of *Chl. chlorochromatii* and "*Ca.* S. mobilis." (A) Two genomic islands, one from each organism, containing mainly genes encoding enzymes involved in cell envelope biosynthesis and other highly similar genes in those regions. (B) A genomic island containing mainly chemotaxis and regulatory genes is very similar to gene clusters in purple sulfur bacteria.

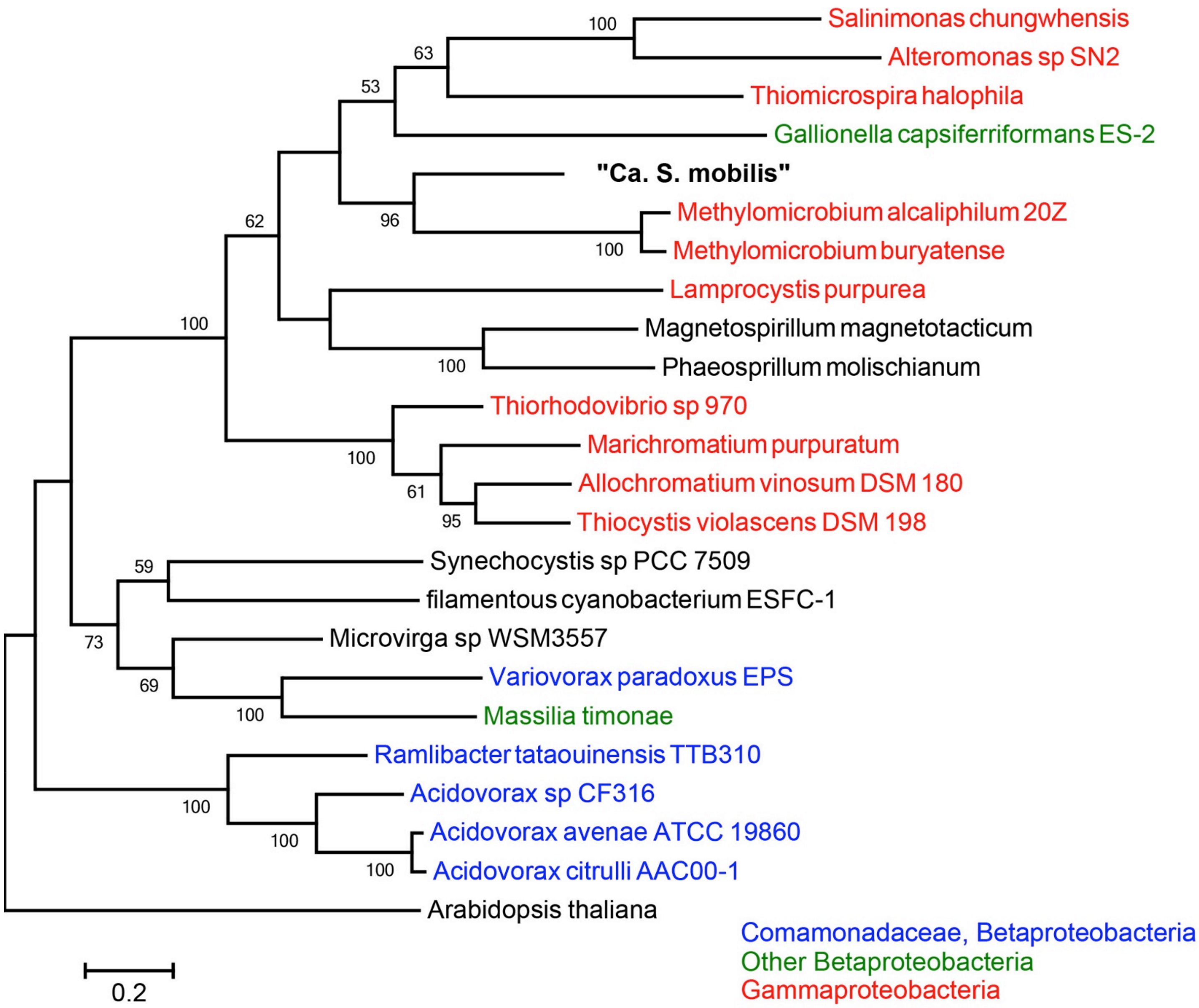

Supplemental Figure S3. Phylogenetic analysis of one bacteriophytochrome gene (Cenrod_2641) and its most similar homologs in the database and those in taxonomically related organisms. Protein sequences were aligned using ClustalW. A maximum likelihood algorithm with JTT model of evolution was used for the phylogenetic analysis. Bootstrap support values of at least 50 among 100 bootstrap samplings are shown. Bar denotes 0.2 changes per amino acid site. The homolog from *Arabidopsis thaliana* was used as outgroup.

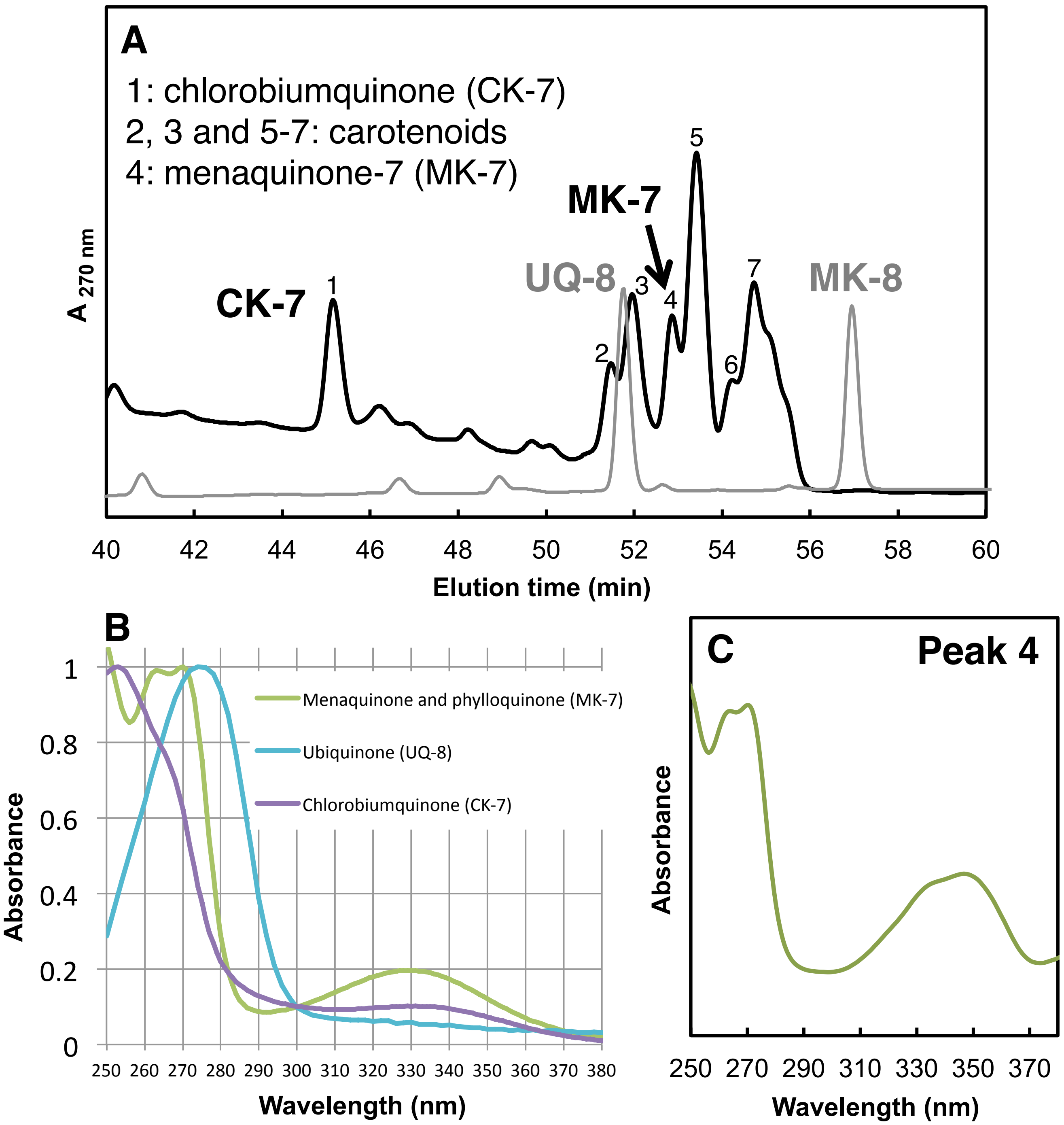

Supplemental Figure S4. Quinone analysis of "*C. aggregatum*." (A) HPLC elution profiles monitored at 270 nm of pigment and quinone extracts from cells. The light gray line shows the elution profile analysis of a solvent extract from a mixture of *Escherichia coli* cells grown under both oxic and anoxic conditions. The elution positions of ubiquinone-8 (UQ-8; 51.5 min) and menaquinone-8 (MK-8; 57 min) are indicated. The black line shows the elution profile of a solvent extract prepared from intact *"C. aggregatum"* consortia. Peaks 2, 4, 5, 6 and 7 had absorption spectra characteristic of carotenoids. Two peaks had absorption properties characteristic of quinones: peak 1 at 45 min was identified as chlorobiumquinone (CK) because it had the same elution time and absorption spectrum (B) as CK from *Chlorobaculum tepidum*. Peak 4, eluting at 53 min, was identified as MK-7 because it co-eluted with MK-7 from *Cba. tepidum* and because of its in-line absorption spectrum (compare panels B and C). The absorption spectrum in Panel C differs from the standard compound in Panel B around 350 nm because of imperfect subtraction of the absorption contribution from the carotenoid (peak 5) in the region of 300 to 400 nm. No other compounds with the absorption properties characteristic of UQ or MK were detected between 40 min and 60 min. This indicates that UQ-6 to UQ-10 as well as MK-6 and MQ-8 do not occur in consortia.
