# Supplementary material for: Genomic analysis reveals key aspects of prokaryotic symbiosis in the phototrophic consortium “Chlorochromatium aggregatum”
Source: Genome Biol. 2013 Nov 22;14(11):R127. doi: 10.1186/gb-2013-14-11-r127 (PMC4053972; doi:10.1186/gb-2013-14-11-r127)
Supplement: Additional file 2: Table S1 — Genes unique to Chl. chlorochromatii that are not found in other GSB. Table S2. Conserved orthologous Comamonadaceae genes that are missing in the genome of “Ca. S. mobilis”. Table S3. Genes unique to “Ca. S. mobilis” that do not have orthologs in 8 other Comamonadaceae genomes. Table S4. Potential horizontal transferred gene pairs of “Ca. S. mobilis” and Chl. chlorochromatii. [file gb-2013-14-11-r127-S2.pdf]

Table S1. Genes unique to *Chl. chlorochromatii* that are not found in other GSB. Orthologs were defined by reciprocal best BLASTP hits with at least 30% amino acid identity and *e*-values smaller than $1e^{-5}$.

Locus tag	Annotation
Cag_0016	conserved hypothetical protein
Cag_0031	hypothetical protein
Cag_0033	hypothetical protein
Cag_0038	hypothetical protein
Cag_0042	hypothetical protein
Cag_0118	hypothetical protein
Cag_0133	Dihydropteroate synthase
Cag_0134	hypothetical protein
Cag_0135	hypothetical protein
Cag_0136	hypothetical protein
Cag_0144	ATPase
Cag_0151	conserved hypothetical protein
Cag_0152	hypothetical protein
Cag_0153	hypothetical protein
Cag_0160	TPR repeat
Cag_0236	putative plasmid maintenance system antidote protein, XRE family
Cag_0253	hypothetical protein
Cag_0256	hypothetical protein
Cag_0266	Protein of unknown function DUF132
Cag_0267	hypothetical protein
Cag_0268	conserved hypothetical protein
Cag_0269	hypothetical protein
Cag_0276	conserved hypothetical protein
Cag_0278	hypothetical protein
Cag_0279	conserved hypothetical protein
Cag_0281	conserved hypothetical protein
Cag_0300	hypothetical protein
Cag_0308	hypothetical protein
Cag_0309	conserved hypothetical protein
Cag_0311	hypothetical protein
Cag_0325	conserved hypothetical protein
Cag_0364	putative signal transduction protein with Nacht domain
Cag_0368	hypothetical protein
Cag_0376	hypothetical protein
Cag_0378	helicase domain protein
Cag_0380	DEAD/DEAH box helicase-like protein
Cag_0396	conserved hypothetical protein
Cag_0424	drug:proton antiporter
Cag_0427	hypothetical protein
Cag_0430	conserved hypothetical protein

Cag_0432	phosphate uptake regulator, PhoU
Cag_0433	arginine decarboxylase
Cag_0434	hypothetical protein
Cag_0445	hypothetical protein
Cag_0507	hypothetical protein
Cag_0513	putative DNA-binding protein
Cag_0519	hypothetical protein
Cag_0529	hypothetical protein
Cag_0532	conserved hypothetical protein
Cag_0535	conserved hypothetical protein
Cag_0544	hypothetical protein
Cag_0545	hypothetical protein
Cag_0546	hypothetical protein
Cag_0553	hypothetical protein
Cag_0554	conserved hypothetical protein
Cag_0555	conserved hypothetical protein
Cag_0561	hypothetical protein
Cag_0576	conserved hypothetical protein
Cag_0577	hypothetical protein
Cag_0578	conserved hypothetical protein
Cag_0581	hypothetical protein
Cag_0582	hypothetical protein
Cag_0584	transcriptional regulator, CopG family
Cag_0585	conserved hypothetical protein
Cag_0599	hypothetical protein
Cag_0600	hypothetical protein
Cag_0614	Parallel beta-helix repeat
Cag_0615	Outer membrane protein-like protein
Cag_0616	Parallel beta-helix repeat
Cag_0646	conserved hypothetical protein F56H9.1
Cag_0648	Periplasmic protein involved in polysaccharide export-like protein
Cag_0649	Uncharacterized protein involved in exopolysaccharide biosynthesis-like protein
Cag_0650	exopolysaccharide biosynthesis protein
Cag_0655	hypothetical protein
Cag_0656	nucleic acid-binding protein, containing PIN domain
Cag_0662	hypothetical protein
Cag_0663	hypothetical protein
Cag_0664	putative transcriptional regulator
Cag_0665	probable polysaccharide biosynthesis protein
Cag_0666	hypothetical protein
Cag_0667	hypothetical protein
Cag_0668	Putative enzyme of poly-gamma-glutamate biosynthesis (capsule formation)-like protein
Cag_0670	conserved hypothetical protein

Cag_0673	putative glycosyltransferase
Cag_0674	hypothetical protein
Cag_0675	putative glycosyl transferase
Cag_0680	hypothetical protein
Cag_0682	transposase
Cag_0683	hypothetical protein
Cag_0686	conserved hypothetical protein
Cag_0690	conserved hypothetical protein
Cag_0691	hypothetical protein
Cag_0696	conserved hypothetical protein
Cag_0697	conserved hypothetical protein
Cag_0705	hypothetical protein
Cag_0706	hypothetical protein
Cag_0707	conserved hypothetical protein
Cag_0709	hypothetical protein
Cag_0714	hypothetical protein
Cag_0718	plasmid segregation centromere-binding protein ParG
Cag_0720	conserved hypothetical protein
Cag_0721	conserved hypothetical protein
Cag_0724	conserved hypothetical protein
Cag_0732	TPR repeat
Cag_0733	conserved hypothetical protein
Cag_0734	hypothetical protein
Cag_0735	hypothetical protein
Cag_0736	hypothetical protein
Cag_0737	nucleotidyltransferase
Cag_0738	VCBS
Cag_0739	hypothetical protein
Cag_0745	glycosyl transferase, family 25
Cag_0746	hypothetical protein
Cag_0749	conserved hypothetical protein
Cag_0750	hypothetical protein
Cag_0751	hypothetical protein
Cag_0755	conserved hypothetical protein
Cag_0759	conserved hypothetical protein
Cag_0760	DEAD/DEAH box helicase-like protein
Cag_0761	conserved hypothetical protein
Cag_0762	hypothetical protein
Cag_0766	conserved hypothetical protein
Cag_0767	possible abortive infection phage resistance protein
Cag_0771	hypothetical protein
Cag_0787	Alkaline phosphatase
Cag_0788	transposase
Cag_0789	periplasmic sensor hybrid histidine kinase
Cag_0790	hypothetical protein

Cag_0818	hypothetical protein
Cag_0819	hypothetical protein
Cag_0834	hypothetical protein
Cag_0835	hypothetical protein
Cag_0840	TPR repeat
Cag_0854	hypothetical protein
Cag_0857	conserved hypothetical protein
Cag_0866	hypothetical protein
Cag_0877	hypothetical protein
Cag_0878	conserved hypothetical protein
Cag_0894	conserved hypothetical protein
Cag_0911	hypothetical protein
Cag_0912	hypothetical protein
Cag_0948	hypothetical protein
Cag_0958	conserved hypothetical protein
Cag_0980	conserved hypothetical protein
Cag_0982	Acyl-CoA synthetases (AMP-forming)/AMP-acid ligases II-like protein
Cag_0984	hypothetical protein
Cag_0985	putative plasmid maintenance system antidote protein, XRE family
Cag_0991	hypothetical protein
Cag_0992	hypothetical protein
Cag_0993	hypothetical protein
Cag_0994	hypothetical protein
Cag_0995	hypothetical protein
Cag_0997	conserved hypothetical protein
Cag_0999	conserved hypothetical protein
Cag_1000	conserved hypothetical protein
Cag_1001	conserved hypothetical protein
Cag_1002	conserved hypothetical protein
Cag_1003	hypothetical protein
Cag_1008	CRISPR-associated protein, Csd1 family
Cag_1009	CRISPR-associated protein, Csd5d family
Cag_1015	conserved hypothetical protein
Cag_1021	conserved hypothetical protein
Cag_1022	hypothetical protein
Cag_1024	conserved hypothetical protein
Cag_1025	conserved hypothetical protein
Cag_1026	C-type lectin
Cag_1034	hypothetical protein
Cag_1036	uncharacterized conserved coiled coil protein
Cag_1046	conserved hypothetical protein
Cag_1047	hypothetical protein
Cag_1048	hypothetical protein
Cag_1050	hypothetical protein
Cag_1051	hypothetical protein

Cag_1053	Filamentous hemagglutinin-like protein
Cag_1055	Filamentous hemagglutinin-like protein
Cag_1056	Hemolysin activation/secretion protein-like protein
Cag_1061	conserved hypothetical phage AbiD protein
Cag_1062	hypothetical protein
Cag_1080	conserved hypothetical protein
Cag_1097	conserved hypothetical protein
Cag_1098	hypothetical protein
Cag_1101	putative transcriptional regulator
Cag_1104	conserved hypothetical protein
Cag_1122	Restriction endonuclease S subunits-like protein
Cag_1123	conserved hypothetical protein
Cag_1131	hypothetical protein
Cag_1147	hypothetical protein
Cag_1178	hypothetical protein
Cag_1191	hypothetical protein
Cag_1196	adenine specific DNA methyltransferase
Cag_1197	conserved hypothetical protein
Cag_1199	conserved hypothetical protein
Cag_1201	hypothetical protein
Cag_1202	conserved hypothetical protein
Cag_1235	hypothetical protein
Cag_1236	hypothetical protein
Cag_1238	hypothetical protein
Cag_1239	VCBS
Cag_1241	conserved hypothetical protein
Cag_1242	VCBS
Cag_1243	hypothetical protein
Cag_1254	hypothetical protein
Cag_1262	conserved hypothetical protein
Cag_1269	hypothetical protein
Cag_1272	hypothetical protein
Cag_1277	hypothetical protein
Cag_1278	hypothetical protein
Cag_1284	hypothetical protein
Cag_1285	hypothetical protein
Cag_1287	hypothetical protein
Cag_1291	hypothetical protein
Cag_1292	conserved hypothetical protein
Cag_1302	ATPase-like protein
Cag_1303	hypothetical protein
Cag_1309	methylase
Cag_1310	HNH nuclease
Cag_1318	hypothetical protein
Cag_1319	transcriptional modulator of MazE/toxin, MazF

Cag_1321	hypothetical protein
Cag_1323	conserved hypothetical protein
Cag_1352	hypothetical protein
Cag_1353	hypothetical protein
Cag_1354	nucleic acid-binding protein contains PIN domain-like protein
Cag_1362	hypothetical protein
Cag_1363	serine/threonine protein kinase
Cag_1364	hypothetical protein
Cag_1373	conserved hypothetical protein
Cag_1374	hypothetical protein
Cag_1386	transcriptional regulator, XRE family
Cag_1387	ATP-dependent endonuclease of the OLD family-like protein
Cag_1389	nucleic acid-binding protein,contains PIN domain
Cag_1390	hypothetical protein
Cag_1391	putative type II restriction enzyme
Cag_1392	putative type II DNA modification enzyme (methyltransferase)
Cag_1401	conserved hypothetical protein
Cag_1406	hypothetical protein
Cag_1407	peptidase, M50 family
Cag_1408	Membrane-fusion protein-like protein
Cag_1409	Membrane-fusion protein-like protein
Cag_1427	hypothetical protein
Cag_1465	hypothetical protein
Cag_1505	hypothetical protein
Cag_1506	conserved hypothetical protein
Cag_1508	hypothetical protein
Cag_1509	conserved hypothetical protein
Cag_1511	hypothetical protein
Cag_1528	restriction modification system, type I
Cag_1529	conserved hypothetical protein
Cag_1530	hypothetical protein
Cag_1534	conserved hypothetical protein
Cag_1535	conserved hypothetical protein
Cag_1537	Elongator protein 3/MiaB/NifB
Cag_1552	hypothetical protein
Cag_1558	conserved hypothetical protein
Cag_1559	transposase for IS1663
Cag_1560	VCBS
Cag_1562	conserved hypothetical protein
Cag_1563	protein translocase subunit secA
Cag_1570	virulence-associated protein D
Cag_1571	hypothetical protein
Cag_1576	hypothetical protein
Cag_1580	transposase
Cag_1597	hypothetical protein

Cag_1598	hypothetical protein
Cag_1599	hypothetical protein
Cag_1600	ATPase
Cag_1606	hypothetical protein
Cag_1609	hypothetical protein
Cag_1610	hypothetical protein
Cag_1611	putative ATPase involved in DNA repair
Cag_1617	conserved hypothetical protein
Cag_1629	conserved hypothetical protein
Cag_1630	hypothetical protein
Cag_1655	FtsK/SpoIIIE family protein
Cag_1671	conserved hypothetical protein
Cag_1690	hypothetical protein
Cag_1697	hypothetical protein
Cag_1698	Outer membrane protein and related peptidoglycan-associated (lipo)proteins-like protein
Cag_1699	hypothetical protein
Cag_1703	hypothetical protein
Cag_1722	hypothetical protein
Cag_1723	hypothetical protein
Cag_1738	hypothetical protein
Cag_1752	conserved hypothetical protein
Cag_1769	Protein of unknown function DUF132
Cag_1770	conserved hypothetical protein
Cag_1772	hypothetical protein
Cag_1794	hypothetical protein
Cag_1795	hypothetical protein
Cag_1796	Ankyrin
Cag_1858	transcriptional regulator, XRE family
Cag_1860	hypothetical protein
Cag_1861	hypothetical protein
Cag_1866	putative DNA-binding protein
Cag_1880	hypothetical protein
Cag_1881	hypothetical protein
Cag_1895	hypothetical protein
Cag_1896	hypothetical protein
Cag_1897	Nidogen, extracellular region
Cag_1904	hypothetical protein
Cag_1909	Cell division and transport-associated protein TolA
Cag_1913	hypothetical protein
Cag_1916	conserved hypothetical protein
Cag_1917	hypothetical protein
Cag_1919	hypothetical protein
Cag_1920	hypothetical protein
Cag_1966	conserved hypothetical protein

Cag_1976	hypothetical protein
Cag_1981	transposase
Cag_1985	Cold shock protein
Cag_2020	hypothetical protein

Table S2. Conserved orthologous *Comamonadaceae* genes that are missing in the genome of "*Ca.* S. mobilis". Orthologs were defined by reciprocal best BLASTP hits with at least 30% amino acid identity and e-values smaller than $1e^{-5}$. Gene conservation is defined by gene presence in eight *Comamonadaceae* organisms with complete genomes, each representing a different genus. Those eight organisms are listed in legend of Figure 2. The locus tags of the missing genes are those for *Rhodoferax ferrireducens* T118.

Locus tag	Annotation
Rfer_0020	hypothetical protein
Rfer_0034	cell division topological specificity factor MinE
Rfer_0048	aldehyde dehydrogenase
Rfer_0052	hypothetical protein
Rfer_0067	hypothetical protein
Rfer_0093	malic enzyme
Rfer_0100	hypothetical protein
Rfer_0113	putative ATP synthase protein I
Rfer_0114	hypothetical protein
Rfer_0121	zinc-binding alcohol dehydrogenase
Rfer_0122	carboxylesterase
Rfer_0158	allantoate amidohydrolase
Rfer_0165	putative bifunctional OHCU decarboxylase/allantoate amidohydrolase
Rfer_0166	acetylornithine deacetylase or succinyl-diaminopimelate desuccinylase
Rfer_0208	class I cytochrome c
Rfer_0213	inner-membrane translocator
Rfer_0296	LysR family transcriptional regulator
Rfer_0326	CreA
Rfer_0332	amidohydrolase 2
Rfer_0341	LysR family transcriptional regulator
Rfer_0372	methylmalonate-semialdehyde dehydrogenase
Rfer_0373	beta alanine--pyruvate transaminase
Rfer_0379	acriflavin resistance protein
Rfer_0380	secretion protein HlyD
Rfer_0401	hypothetical protein
Rfer_0403	peptidase M20
Rfer_0422	two component heavy metal response transcriptional regulator
Rfer_0441	LysR family transcriptional regulator
Rfer_0447	GntR-like protein
Rfer_0450	TRAP dicarboxylate transporter subunit DctP
Rfer_0457	LysR family transcriptional regulator
Rfer_0480	glycolate oxidase FAD-binding subunit
Rfer_0481	glycolate oxidase iron-sulfur subunit
Rfer_0520	hypothetical protein
Rfer_0523	acyl-CoA dehydrogenase-like protein
Rfer_0525	phospholipase
Rfer_0531	ApaG protein
Rfer_0576	serine/threonine protein kinase

Rfer_0598	succinic semialdehyde dehydrogenase
Rfer_0606	FAD linked oxidase-like protein
Rfer_0608	enoyl-CoA hydratase/isomerase
Rfer_0610	hypothetical protein
Rfer_0613	fumarylacetoacetate (FAA) hydrolase
Rfer_0617	hypothetical protein
Rfer_0621	hypothetical protein
Rfer_0622	hypothetical protein
Rfer_0642	ABC transporter-like protein
Rfer_0731	putative ubiquinone biosynthesis monooxygenase COQ7
Rfer_0732	OsmC-like protein
Rfer_0736	ExsB
Rfer_0753	ferric uptake regulator family protein
Rfer_0763	glutamate--cysteine ligase GshA
Rfer_0765	glutathione synthetase
Rfer_0799	type II secretion system protein E
Rfer_0814	dihydroneopterin aldolase
Rfer_0815	tRNA 2-thiocytidine biosynthesis protein TtcA
Rfer_0816	phosphoglycerate mutase
Rfer_0849	Nitrilase/cyanide hydratase and apolipoprotein N-acyltransferase
Rfer_0853	MerR family transcriptional regulator
Rfer_0866	hypothetical protein
Rfer_0890	IclR family transcriptional regulator
Rfer_0922	multi-sensor signal transduction histidine kinase
Rfer_0941	2-dehydro-3-deoxyglucarate aldolase
Rfer_0957	LacI family transcription regulator
Rfer_0974	phenylacetic acid degradation-like protein
Rfer_0995	enoyl-CoA hydratase/isomerase
Rfer_1008	2-nitropropane dioxygenase
Rfer_1027	galactarate dehydratase
Rfer_1035	3-oxoacid CoA-transferase subunit B
Rfer_1036	3-oxoacid CoA-transferase subunit A
Rfer_1075	ABC transporter-like protein
Rfer_1076	ABC transporter-like protein
Rfer_1077	extracellular ligand-binding receptor
Rfer_1078	inner-membrane translocator
Rfer_1079	inner-membrane translocator
Rfer_1086	LysR family transcriptional regulator
Rfer_1089	glutathione S-transferase-like protein
Rfer_1290	hypothetical protein
Rfer_1307	UBA/THIF-type NAD/FAD-binding protein
Rfer_1311	glutaredoxin GrxC
Rfer_1314	cyanophycin synthetase
Rfer_1315	cyanophycin synthetase
Rfer_1316	ABC transporter-like protein

Rfer_1317	hypothetical protein
Rfer_1335	isocitrate lyase
Rfer_1346	cyclophilin type peptidyl-prolyl cis-trans isomerase
Rfer_1370	rubredoxin-type Fe(Cys)4 protein
Rfer_1388	GTP-binding
Rfer_1400	hypothetical protein
Rfer_1406	NUDIX hydrolase
Rfer_1410	transcriptional coactivator/pterin dehydratase
Rfer_1429	zinc-binding alcohol dehydrogenase
Rfer_1434	HNH endonuclease
Rfer_1440	C4-dicarboxylate transporter DctA
Rfer_1466	MoeA-like proteindomain-containing protein
Rfer_1467	molybdopterin-guanine dinucleotide biosynthesis protein MobA
Rfer_1468	molybdenum cofactor biosynthesis protein A
Rfer_1473	inorganic diphosphatase
Rfer_1477	FAD linked oxidase-like protein
Rfer_1508	hypothetical protein
Rfer_1514	RND efflux system outer membrane lipoprotein NodT
Rfer_1532	biopolymer transport protein ExbD/TolR
Rfer_1573	enoyl-CoA hydratase/isomerase
Rfer_1574	peptidase M61
Rfer_1576	UbiH/UbiF/VisC/COQ6 family ubiquinone biosynthesis hydroxylase
Rfer_1579	hypothetical protein
Rfer_1580	dihydrouridine synthase TIM-barrel protein nifR3
Rfer_1588	class I cytochrome c
Rfer_1589	AAA_5 ATPase
Rfer_1590	GCN5-like protein N-acetyltransferase
Rfer_1591	hypothetical protein
Rfer_1592	surface antigen (D15)
Rfer_1593	hypothetical protein
Rfer_1594	binding-protein-dependent transport systems inner membrane component
Rfer_1595	ABC transporter-like protein
Rfer_1645	acyl-CoA dehydrogenase-like protein
Rfer_1646	acyl-CoA dehydrogenase-like protein
Rfer_1648	hypothetical protein
Rfer_1656	formamidopyrimidine-DNA glycosylase
Rfer_1664	4Fe-4S ferredoxin
Rfer_1670	peptidase M16-like protein
Rfer_1671	peptidase M16-like protein
Rfer_1680	cytochrome c oxidase subunit II
Rfer_1681	cytochrome-c oxidase
Rfer_1682	cytochrome C oxidase assembly protein
Rfer_1684	cytochrome c oxidase subunit III
Rfer_1686	hypothetical protein
Rfer_1687	hypothetical protein

Rfer_1688	cytochrome oxidase assembly
Rfer_1689	protoheme IX farnesyltransferase
Rfer_1690	electron transport protein SCO1/SenC
Rfer_1691	hypothetical protein
Rfer_1700	nitrogen regulatory protein P-II (GlnB, GlnK)
Rfer_1708	thiamine biosynthesis protein ThiC
Rfer_1711	histidine kinase internal protein
Rfer_1712	LytR/AlgR family transcriptional regulator
Rfer_1714	phosphoenolpyruvate carboxylase
Rfer_1722	HAD family hydrolase
Rfer_1723	Rieske (2Fe-2S) protein
Rfer_1736	RNA polymerase sigma factor RpoE
Rfer_1759	sulfate transport system permease 1
Rfer_1760	Sulfate ABC transporter permease CysW
Rfer_1761	Sulfate ABC transporter permease CysT
Rfer_1762	thiosulfate-binding protein
Rfer_1781	thiosulfate-binding protein
Rfer_1791	peptidoglycan-binding LysM
Rfer_1802	GntR family transcriptional regulator
Rfer_1805	hypothetical protein
Rfer_1828	peptidase S16, lon-like protein
Rfer_1867	D-3-phosphoglycerate dehydrogenase
Rfer_1872	response regulator receiver domain-containing protein
Rfer_1873	two component LuxR family transcriptional regulator
Rfer_1874	histidine kinase
Rfer_1895	pseudouridine synthase
Rfer_1905	glutamate--tRNA ligase
Rfer_1906	FAD dependent oxidoreductase
Rfer_1908	PhoH-like protein
Rfer_1915	MoeA-like proteindomain-containing protein
Rfer_1916	molybdopterin converting factor subunit 1
Rfer_1917	molybdopterin biosynthesis MoaE
Rfer_1922	LysR family transcriptional regulator
Rfer_1925	malonyl-CoA synthase
Rfer_1946	major facilitator transporter
Rfer_1951	oligopeptide/dipeptide ABC transporter ATP-binding protein-like protein
Rfer_1954	SWIB/MDM2
Rfer_1959	acyl-CoA dehydrogenase-like protein
Rfer_1960	2-nitropropane dioxygenase
Rfer_1974	polyhydroxyalkanoate synthesis repressor PhaR
Rfer_1988	amidase
Rfer_2003	tRNA/rRNA methyltransferase SpoU
Rfer_2008	hypothetical protein
Rfer_2021	phospholipid/glycerol acyltransferase
Rfer_2028	hypothetical protein

Rfer_2049	malonyl-CoA decarboxylase
Rfer_2060	phosphogluconate dehydratase
Rfer_2061	2-dehydro-3-deoxyphosphogluconate aldolase/4-hydroxy-2-oxoglutarate aldolase
Rfer_2064	RND efflux system outer membrane lipoprotein NodT
Rfer_2069	3-hydroxybutyrate dehydrogenase
Rfer_2070	alpha/beta hydrolase
Rfer_2073	uracil phosphoribosyltransferase
Rfer_2077	coproporphyrinogen III oxidase
Rfer_2081	Maf-like protein
Rfer_2098	hypothetical protein
Rfer_2099	major facilitator transporter
Rfer_2101	FAD dependent oxidoreductase
Rfer_2111	putative aminotransferase
Rfer_2112	transcriptional regulator CysB-like protein
Rfer_2113	cobalamin (vitamin B12) biosynthesis CbiX protein
Rfer_2139	3-hydroxyisobutyryl-CoA hydrolase
Rfer_2140	phasin
Rfer_2141	thioesterase superfamily protein
Rfer_2144	D-isomer specific 2-hydroxyacid dehydrogenase
Rfer_2169	DEAD/DEAH box helicase-like protein
Rfer_2190	hypothetical protein
Rfer_2191	phosphoenolpyruvate synthase
Rfer_2199	chromosome segregation protein SMC
Rfer_2202	peptide deformylase
Rfer_2204	short-chain dehydrogenase/reductase SDR
Rfer_2211	multi-sensor signal transduction histidine kinase
Rfer_2214	dihydrolipoamide dehydrogenase
Rfer_2235	hypothetical protein
Rfer_2247	tartrate dehydrogenase
Rfer_2290	hypothetical protein
Rfer_2295	phosphoribosyltransferase
Rfer_2313	putative transcriptional regulator
Rfer_2316	helicase c2
Rfer_2322	propionyl-CoA synthetase
Rfer_2323	isochorismatase hydrolase
Rfer_2329	hydroxypyruvate reductase
Rfer_2340	polyhydroxyalkanoate depolymerase
Rfer_2341	RnfABCDGE type electron transport complex subunit B
Rfer_2347	IclR family transcriptional regulator
Rfer_2348	arginyl-tRNA-protein transferase
Rfer_2351	L-lactate dehydrogenase (cytochrome)
Rfer_2353	DNA-directed DNA polymerase
Rfer_2365	fumarate hydratase
Rfer_2366	aconitate hydratase

Rfer_2370	7-cyano-7-deazaguanine reductase
Rfer_2378	cysteine synthase B
Rfer_2383	alpha/beta hydrolase fold-3 protein
Rfer_2385	acetyl-CoA acetyltransferase
Rfer_2386	3-hydroxyacyl-CoA dehydrogenase
Rfer_2393	long-chain-fatty-acid--CoA ligase
Rfer_2396	protein-methionine-S-oxide reductase
Rfer_2398	BolA-like protein
Rfer_2417	cyclopropane-fatty-acyl-phospholipid synthase
Rfer_2424	lipolytic enzyme, G-D-S-L
Rfer_2425	ABC transporter-like protein
Rfer_2456	nitrite and sulfite reductase 4Fe-4S protein
Rfer_2457	hypothetical protein
Rfer_2459	sulfate adenylyltransferase subunit 2
Rfer_2460	Sulfate adenylyltransferase large subunit
Rfer_2462	FAD-dependent pyridine nucleotide-disulfide oxidoreductase
Rfer_2464	FAD dependent oxidoreductase
Rfer_2466	thiazole synthase
Rfer_2467	thiamine-phosphate diphosphorylase
Rfer_2469	hypothetical protein
Rfer_2483	RNA-binding S1
Rfer_2491	zinc-binding alcohol dehydrogenase
Rfer_2492	phenylacetic acid degradation-like protein
Rfer_2493	gluconate 5-dehydrogenase
Rfer_2526	hypothetical protein
Rfer_2528	histone deacetylase superfamily protein
Rfer_2552	glyoxalase I
Rfer_2557	nitrite reductase (NAD(P)H) large subunit NirB
Rfer_2559	molybdopterin oxidoreductase
Rfer_2560	3-ketoacyl-(acyl-carrier-protein) reductase
Rfer_2561	acetyl-CoA acetyltransferase
Rfer_2562	poly(R)-hydroxyalkanoic acid synthase, class I
Rfer_2567	fumarylacetoacetate (FAA) hydrolase
Rfer_2571	NUDIX hydrolase
Rfer_2581	acetate--CoA ligase
Rfer_2598	peptide methionine sulfoxide reductase
Rfer_2603	rhodanese-like protein
Rfer_2612	cobyric acid synthase CobQ
Rfer_2618	cob(I)alamin adenosyltransferase
Rfer_2634	3-oxoacid CoA-transferase subunit B
Rfer_2635	3-oxoacid CoA-transferase subunit A
Rfer_2644	cupin 4
Rfer_2646	deoxyuridine 5'-triphosphate nucleotidohydrolase
Rfer_2655	auxin efflux carrier
Rfer_2664	methylation

Rfer_2687	hypothetical protein
Rfer_2697	aldo/keto reductase
Rfer_2704	fructose-1,6-bisphosphatase
Rfer_2745	L-carnitine dehydratase/bile acid-inducible protein F
Rfer_2756	chromate transporter
Rfer_2757	chromate transporter
Rfer_2758	extracellular solute-binding protein
Rfer_2769	GCN5-like protein N-acetyltransferase
Rfer_2775	hypothetical protein
Rfer_2781	protein-L-isoaspartate O-methyltransferase
Rfer_2783	zinc-binding alcohol dehydrogenase
Rfer_2801	lactoylglutathione lyase
Rfer_2802	carbamoyl-phosphate synthase L chain, ATP-binding
Rfer_2803	propionyl-CoA carboxylase
Rfer_2804	arginine/ornithine transport system ATPase
Rfer_2805	methylmalonyl-CoA mutase
Rfer_2806	GntR family transcriptional regulator
Rfer_2811	trifunctional transcriptional regulator/proline dehydrogenase/pyrroline-5-carboxylate dehydrogenase
Rfer_2865	iron-containing alcohol dehydrogenase
Rfer_2866	aldehyde dehydrogenase
Rfer_2871	tripartite ATP-independent periplasmic transporter DctQ
Rfer_2872	TRAP dicarboxylate transporter subunit DctM
Rfer_2878	Rieske (2Fe-2S) protein
Rfer_2879	hypothetical protein
Rfer_2880	3-mercaptopyruvate sulfurtransferase
Rfer_2882	glycosyl transferase family protein
Rfer_2883	uroporphyrin-III C-methyltransferase
Rfer_2888	hypothetical protein
Rfer_2889	molybdenum cofactor biosynthesis protein MogA
Rfer_2908	NUDIX hydrolase
Rfer_2909	hypothetical protein
Rfer_2930	hypothetical protein
Rfer_2932	hypothetical protein
Rfer_2940	sulfate transporter/antisigma-factor antagonist STAS
Rfer_2941	ABC transporter-like protein
Rfer_2943	BolA-like protein
Rfer_2956	twin arginine translocase protein A
Rfer_2961	hypothetical protein
Rfer_2964	ubiquinol-cytochrome c reductase, iron-sulfur subunit
Rfer_2965	cytochrome b/b6-like protein
Rfer_2966	cytochrome c1
Rfer_2990	membrane protein-like protein
Rfer_2996	D-isomer specific 2-hydroxyacid dehydrogenase
Rfer_3029	hypothetical protein

Rfer_3030	chromosome segregation and condensation protein ScpA
Rfer_3133	5-oxopent-3-ene-1,2,5-tricarboxylate decarboxylase
Rfer_3142	hypothetical protein
Rfer_3159	alpha/beta hydrolase
Rfer_3160	O-acetylhomoserine aminocarboxypropyltransferase
Rfer_3164	inner-membrane translocator
Rfer_3165	inner-membrane translocator
Rfer_3166	ABC transporter-like protein
Rfer_3183	Fmu (Sun)
Rfer_3191	class I cytochrome c
Rfer_3195	short-chain dehydrogenase/reductase SDR
Rfer_3212	hypothetical protein
Rfer_3213	hypothetical protein
Rfer_3233	hypothetical protein
Rfer_3255	hypothetical protein
Rfer_3297	adenosine deaminase
Rfer_3298	NAD-dependent epimerase/dehydratase
Rfer_3300	hypothetical protein
Rfer_3318	hypothetical protein
Rfer_3326	Crp/FNR family transcriptional regulator
Rfer_3327	aminoglycoside phosphotransferase
Rfer_3328	acyl-CoA dehydrogenase-like protein
Rfer_3343	cytochrome c assembly protein
Rfer_3347	ribonucleotide-diphosphate reductase subunit alpha
Rfer_3348	ribonucleotide-diphosphate reductase subunit beta
Rfer_3354	putative thioredoxin-like protein transmembrane protein
Rfer_3355	hypothetical protein
Rfer_3386	gamma-glutamyltransferase
Rfer_3387	negative transcriptional regulator
Rfer_3432	FtsL-like protein cell division protein
Rfer_3446	beta-lactamase-like protein
Rfer_3457	RNA-binding S4
Rfer_3465	LysR family transcriptional regulator
Rfer_3472	hypothetical protein
Rfer_3479	LysR family transcriptional regulator
Rfer_3493	ABC transporter-like protein
Rfer_3512	enoyl-CoA hydratase
Rfer_3519	acyl-CoA dehydrogenase-like protein
Rfer_3520	TetR family transcriptional regulator
Rfer_3536	putative phenylacetate-CoA ligase
Rfer_3537	ABC transporter-like protein
Rfer_3538	hypothetical protein
Rfer_3539	inner-membrane translocator
Rfer_3540	inner-membrane translocator
Rfer_3541	ABC transporter-like protein

Rfer_3542	AMP-dependent synthetase and ligase
Rfer_3544	RNA-binding S4
Rfer_3569	AMP nucleosidase
Rfer_3580	beta-lactamase-like protein
Rfer_3581	malate synthase G
Rfer_3613	rhodanese-like protein
Rfer_3615	peptidase M20D, amidohydrolase
Rfer_3648	AsnC family transcriptional regulator
Rfer_3678	glycerol kinase
Rfer_3699	GCN5-like protein N-acetyltransferase
Rfer_3729	class I/II aminotransferase
Rfer_3732	phosphoglycerate mutase
Rfer_3752	AMP-dependent synthetase and ligase
Rfer_3784	GCN5-like protein N-acetyltransferase
Rfer_3785	alkyl hydroperoxide reductase/ Thiol specific antioxidant/ Mal allergen
Rfer_3802	ferredoxin-like protein
Rfer_3803	VanZ-like protein
Rfer_3804	hypothetical protein
Rfer_3806	Mg2+ transporter protein, CorA-like protein
Rfer_3810	carboxymethylenebutenolidase
Rfer_3814	excinuclease ABC subunit A
Rfer_3815	extracellular ligand-binding receptor
Rfer_3816	DSBA oxidoreductase
Rfer_3823	YbaK/prolyl-tRNA synthetase associated protein
Rfer_3824	pyruvate carboxyltransferase
Rfer_3829	D-isomer specific 2-hydroxyacid dehydrogenase
Rfer_3830	carbamoyl-phosphate synthase L chain, ATP-binding
Rfer_3831	enoyl-CoA hydratase
Rfer_3832	propionyl-CoA carboxylase
Rfer_3833	AMP-binding domain-containing protein
Rfer_3838	acyl-CoA dehydrogenase-like protein
Rfer_3839	Acetyl-CoA C-acetyltransferase
Rfer_3841	carbonate dehydratase
Rfer_3842	acyl-CoA dehydrogenase-like protein
Rfer_3868	indolepyruvate ferredoxin oxidoreductase
Rfer_3876	6-phosphogluconate dehydrogenase
Rfer_3884	arginase
Rfer_3914	putative lipoprotein
Rfer_3915	hypothetical protein
Rfer_3916	ABC transporter-like protein
Rfer_3917	hypothetical protein
Rfer_3918	glucose-methanol-choline oxidoreductase
Rfer_3936	hypothetical protein
Rfer_3943	integral membrane protein TerC
Rfer_3949	transcriptional regulatory protein

Rfer_3950	two component transcriptional regulator
Rfer_3951	periplasmic sensor signal transduction histidine kinase
Rfer_3954	nuclear protein SET
Rfer_3963	acetoacetyl-CoA synthetase
Rfer_4040	molybdenum cofactor biosynthesis protein C
Rfer_4042	hypothetical protein
Rfer_4045	hypothetical protein
Rfer_4048	YaeQ
Rfer_4057	flavin reductase-like protein
Rfer_4069	L-carnitine dehydratase/bile acid-inducible protein F
Rfer_4070	acyl-CoA dehydrogenase-like protein
Rfer_4113	NADH:flavin oxidoreductase/NADH oxidase
Rfer_4155	N-formylglutamate amidohydrolase

Table S3. Genes unique to "*Ca.* S. mobilis" that do not have orthologs in 8 other *Comamonadaceae* genomes. Orthologs were defined by reciprocal best BLASTP hits with at least 30% amino acid identity and *e*-values smaller than $1e^{-5}$. 8 *Comamonadaceae* organisms were listed in the legend for Figure 2. 1055 such genes were found, but only 444 of them that have putative annotated functions are listed here. The others are annotated as hypothetical proteins.

Locus tag	Annotation
Cenrod_0004	DNA polymerase subunit beta
Cenrod_0006	integrase catalytic subunit
Cenrod_0008	methyltransferase type 11
Cenrod_0014	methyltransferase type 12
Cenrod_0017	glycosyltransferase-like protein
Cenrod_0048	SAM-dependent methyltransferase
Cenrod_0050	helix-turn-helix protein CopG
Cenrod_0051	plasmid-related protein
Cenrod_0057	GTPase-like protein
Cenrod_0066	retron-type reverse transcriptase
Cenrod_0072	PilT domain protein
Cenrod_0073	transposase
Cenrod_0076	cytotoxic translational repressor
Cenrod_0077	transcriptional regulator-like protein
Cenrod_0078	transposase
Cenrod_0082	retron-type reverse transcriptase
Cenrod_0095	methyl-accepting chemotaxis protein
Cenrod_0096	methyl-accepting chemotaxis protein
Cenrod_0097	methyl-accepting chemotaxis protein
Cenrod_0101	group II intron maturase-specific domain protein
Cenrod_0105	integron integrase
Cenrod_0110	dinitrogenase iron-molybdenum cofactor biosynthesis-like protein
Cenrod_0133	ABC-type peptide/nickel transporter substrate-binding protein
Cenrod_0145	signal transduction protein
Cenrod_0146	signal transduction protein
Cenrod_0154	site-specific DNA-methyltransferase
Cenrod_0155	type II restriction enzyme
Cenrod_0159	response regulator
Cenrod_0169	signal transduction histidine kinase
Cenrod_0170	GGDEF domain protein
Cenrod_0177	CheY-like chemotaxis protein
Cenrod_0188	transposase
Cenrod_0210	exoprotein
Cenrod_0212	hemolysin activation/secretion protein
Cenrod_0213	exoprotein
Cenrod_0233	repeat-containing PBS lyase heat domain protein
Cenrod_0235	TPR repeat protein

Cenrod_0237	WD40 repeat protein
Cenrod_0242	IS4 family transposase
Cenrod_0247	TrkA-N domain protein
Cenrod_0250	NTPase-like protein
Cenrod_0254	NTPase-like protein
Cenrod_0259	trypsin-like serine protease
Cenrod_0280	permease-like protein
Cenrod_0328	methyl-accepting chemotaxis protein
Cenrod_0331	methyl-accepting chemotaxis protein
Cenrod_0342	response regulator
Cenrod_0346	GGDEF domain protein
Cenrod_0353	xylanase/chitin deacetylase
Cenrod_0357	cellulose biosynthesis protein BcsE
Cenrod_0401	signal transduction protein
Cenrod_0435	calcium-binding EF-hand-like protein
Cenrod_0437	EAL domain protein
Cenrod_0438	signal transduction protein
Cenrod_0440	NtrC family signal transduction histidine kinase
Cenrod_0450	PIN domain nucleic acid-binding-like protein
Cenrod_0451	sporulation regulator-like protein
Cenrod_0458	DNA/RNA SNF2 family helicase
Cenrod_0474	colanic acid biosynthesis glycosyl-transferase
Cenrod_0475	transposase
Cenrod_0479	UDP-N-acetylglucosamine 2-epimerase
Cenrod_0480	dTDP-4-dehydrorhamnose 3,5-epimerase-like protein
Cenrod_0482	XRE family transcriptional regulator
Cenrod_0484	lambda repressor-like DNA-binding protein
Cenrod_0485	colanic acid biosynthesis glycosyl-transferase
Cenrod_0487	transposase-like protein
Cenrod_0491	dTDP-4-dehydrorhamnose 3,5-epimerase-like protein
Cenrod_0493	dTDP-4-dehydrorhamnose 3,5-epimerase-like protein
Cenrod_0494	glycosyltransferase
Cenrod_0499	nucleic-acid-binding-like protein
Cenrod_0503	cell wall biogenesis glycosyltransferase
Cenrod_0504	SAM-dependent methyltransferase
Cenrod_0505	glycosyltransferase
Cenrod_0508	glycosyltransferase
Cenrod_0510	isoprenoid quinone biosynthesis methyltransferase-like protein
Cenrod_0522	nucleoside-diphosphate-sugar epimerase
Cenrod_0523	nucleoside-diphosphate-sugar epimerase
Cenrod_0545	signal transduction histidine kinase
Cenrod_0556	diguanylate cyclase
Cenrod_0564	metal-dependent phosphohydrolase
Cenrod_0569	outer membrane protein
Cenrod_0570	bifunctional chitinase/lysozyme

Cenrod_0581	DNA/RNA SNF2 family helicase
Cenrod_0582	type II restriction enzyme methyltransferase subunit
Cenrod_0597	transcriptional regulator
Cenrod_0609	PilT domain protein
Cenrod_0647	HNH nuclease
Cenrod_0649	ATPase-like protein
Cenrod_0680	membrane protein
Cenrod_0681	ABC-type transporter component
Cenrod_0682	outer membrane protein
Cenrod_0683	calcium-binding RTX toxin-like protein
Cenrod_0693	cellobiose phosphorylase
Cenrod_0695	signal transduction histidine kinase SLN1
Cenrod_0699	methyl-accepting chemotaxis protein
Cenrod_0700	methyl-accepting chemotaxis protein
Cenrod_0707	transcriptional regulator
Cenrod_0708	plasmid stabilization protein
Cenrod_0711	KilA domain protein
Cenrod_0717	transposase
Cenrod_0803	hydrolase-like protein
Cenrod_0812	CRISPR-associated NE0113-family protein
Cenrod_0814	hydrolase-like protein
Cenrod_0817	CRISPR-associated protein Cmr5
Cenrod_0818	DNA repair protein
Cenrod_0821	CRISPR-associated protein Cas2
Cenrod_0823	CRISPR-associated protein Cas2
Cenrod_0827	CRISPR-associated NE0113-family protein
Cenrod_0830	CRISPR-associated NE0113-family protein
Cenrod_0836	integrase catalytic subunit
Cenrod_0841	adenosine deaminase
Cenrod_0844	signal transduction histidine kinase
Cenrod_0848	extracellular protein
Cenrod_0851	signal transduction protein
Cenrod_0852	rhodanese domain protein
Cenrod_0853	methyl-accepting chemotaxis protein
Cenrod_0856	calcium-binding RTX toxin-like protein
Cenrod_0858	signal transduction histidine kinase
Cenrod_0866	DNA/RNA SNF2 family helicase
Cenrod_0867	nucleic-acid-binding-like protein
Cenrod_0869	DNA methylase
Cenrod_0873	ATPase-like protein
Cenrod_0878	DNA polymerase subunit III
Cenrod_0940	transposase
Cenrod_0941	transposase
Cenrod_0957	transposase
Cenrod_0963	transposase

Cenrod_0965	phosphoesterase-like protein
Cenrod_0969	4-hydroxybenzoate polyprenyl transferase
Cenrod_0970	histone acetyltransferase-like protein
Cenrod_1001	NarL family signal transduction histidine kinase
Cenrod_1013	NarL family signal transduction histidine kinase
Cenrod_1029	permease-like protein
Cenrod_1045	elongation factor EF-Tu
Cenrod_1064	methyl-accepting chemotaxis protein
Cenrod_1070	signal transduction histidine kinase
Cenrod_1071	HD-GYP domain protein
Cenrod_1073	signal transduction histidine kinase
Cenrod_1081	type IV pilus modification protein PilV
Cenrod_1084	type IV pilus assembly protein PilY1
Cenrod_1126	signal transduction histidine kinase
Cenrod_1144	prevent-host-death family protein
Cenrod_1152	bacteriophytochrome
Cenrod_1155	methyl-accepting chemotaxis protein
Cenrod_1178	membrane protein
Cenrod_1181	calcium-binding RTX toxin-like protein
Cenrod_1189	methyl-accepting chemotaxis protein
Cenrod_1190	chemotaxis protein CheW
Cenrod_1192	repeat-containing PBS lyase heat domain protein
Cenrod_1193	chemotaxis methyltransferase CheR
Cenrod_1194	chemotaxic protein CheY
Cenrod_1195	chemotaxis histidine kinase CheA
Cenrod_1197	chemotaxic protein CheY
Cenrod_1198	methyl-accepting chemotaxis protein
Cenrod_1201	anti-sigma regulatory factor
Cenrod_1203	protein serine/threonine phosphatase
Cenrod_1204	signal transduction histidine kinase
Cenrod_1205	CheY-like chemotaxis protein
Cenrod_1206	chromosome partitioning protein
Cenrod_1211	saccharopine dehydrogenase-like protein
Cenrod_1224	methyl-accepting chemotaxis protein
Cenrod_1225	GGDEF domain protein
Cenrod_1244	nucleotidyltransferase substrate-binding protein
Cenrod_1262	O-antigen polymerase
Cenrod_1266	ABC-type transporter periplasmic subunit
Cenrod_1273	DNA methylase
Cenrod_1277	N6 DNA methylase
Cenrod_1278	bacteriophage resistance-like protein
Cenrod_1291	type I restriction-modification system methyltransferase subunit
Cenrod_1307	type I restriction enzyme subunit S
Cenrod_1311	transposase
Cenrod_1326	transcriptional regulator

Cenrod_1330	type III restriction protein res subunit
Cenrod_1334	PIN domain nucleic acid-binding-like protein
Cenrod_1336	RNA helicase-like protein
Cenrod_1337	transcriptional regulator
Cenrod_1351	DNA segregation protein
Cenrod_1352	signal transduction protein
Cenrod_1353	growth inhibitor-like protein
Cenrod_1354	transcriptional regulator
Cenrod_1356	UDP-N-acetyl-D-mannosaminuronic acid transferase
Cenrod_1357	glycosyltransferase
Cenrod_1358	acetyltransferase
Cenrod_1359	glycosidase-like protein
Cenrod_1363	cell cycle response regulator
Cenrod_1367	O-antigen polymerase
Cenrod_1368	membrane-bound exporter protein
Cenrod_1371	protein-tyrosine kinase
Cenrod_1372	exopolysaccharide biosynthesis protein
Cenrod_1373	polysaccharide exporter
Cenrod_1399	nucleoside-diphosphate-sugar epimerase-like protein
Cenrod_1400	sialic acid synthase
Cenrod_1405	transposase-like protein
Cenrod_1408	methyl-accepting chemotaxis protein
Cenrod_1409	LasA family protein
Cenrod_1419	DEAD/DEAH box helicase-like protein
Cenrod_1423	helix-hairpin-helix DNA-binding motif-containing protein
Cenrod_1425	transcriptional regulator
Cenrod_1426	addiction module antidote-like protein
Cenrod_1429	LasA family protein
Cenrod_1432	lipoprotein
Cenrod_1455	type II restriction enzyme methyltransferase subunit
Cenrod_1461	phosphoglucomutase
Cenrod_1477	signal transduction histidine kinase
Cenrod_1490	fimbrial protein
Cenrod_1499	homospermidine synthase
Cenrod_1503	chemotaxis methyltransferase CheR
Cenrod_1504	chemotaxis methyltransferase protein
Cenrod_1506	adenylate cyclase
Cenrod_1514	transcriptional regulator
Cenrod_1517	transcriptional regulator
Cenrod_1525	response regulator-like protein
Cenrod_1529	GGDEF domain protein
Cenrod_1536	signal transduction histidine kinase
Cenrod_1538	response regulator-like protein
Cenrod_1539	signal transduction histidine kinase
Cenrod_1540	signal transduction protein

Cenrod_1541	type III HopAG1 effector
Cenrod_1552	cell division protein
Cenrod_1553	transcriptional regulator
Cenrod_1556	transposase
Cenrod_1570	resolvase-like protein
Cenrod_1590	chromosome partitioning type protein
Cenrod_1591	cellulose synthase-like protein
Cenrod_1594	glycosylhydrolase-like protein
Cenrod_1597	signal transduction protein
Cenrod_1610	lipid A core-like protein
Cenrod_1617	exo-beta-1,3-glucanase
Cenrod_1619	glycosylhydrolase-like protein
Cenrod_1620	beta-glucan synthetase
Cenrod_1628	PAS/PAC domain protein
Cenrod_1629	sugar transporter substrate-binding protein
Cenrod_1631	signal transduction protein
Cenrod_1646	serine/threonine protein phosphatase
Cenrod_1653	DnaJ/HSP40 domain protein
Cenrod_1657	transposase
Cenrod_1660	transposase
Cenrod_1661	transposase
Cenrod_1666	cobalamin biosynthesis-like protein
Cenrod_1668	transposase
Cenrod_1669	antitoxin-related protein
Cenrod_1673	PemK-like protein
Cenrod_1680	signal transduction protein
Cenrod_1681	signal transduction histidine kinase
Cenrod_1682	membrane protease subunit
Cenrod_1688	DNA methyltransferase-like protein
Cenrod_1711	general secretion pathway protein F
Cenrod_1715	TPR repeat protein
Cenrod_1717	retron-type reverse transcriptase
Cenrod_1721	retron-type reverse transcriptase
Cenrod_1726	integron/retron-type RNA-directed DNA polymerase
Cenrod_1740	transcriptional regulator-like protein
Cenrod_1741	ATPase-like protein
Cenrod_1746	kinase-like protein
Cenrod_1747	signal transduction histidine kinase
Cenrod_1749	cellobiose phosphorylase
Cenrod_1766	glycosyltransferase
Cenrod_1767	cell wall biogenesis glycosyltransferase
Cenrod_1780	CheY-like chemotaxis protein
Cenrod_1783	chemotaxic protein CheY
Cenrod_1784	anti-sigma-factor antagonist
Cenrod_1785	chemotaxis histidine kinase CheA

Cenrod_1786	extracellular protein
Cenrod_1787	methyl-accepting chemotaxis protein
Cenrod_1788	chemotaxis protein CheW
Cenrod_1790	methyl-accepting chemotaxis protein
Cenrod_1791	chemotaxis signal transduction protein
Cenrod_1793	methyl-accepting chemotaxis protein
Cenrod_1794	response regulator-like protein
Cenrod_1798	GGDEF domain protein
Cenrod_1799	methyltransferase FkbM
Cenrod_1802	CDP-6-deoxy-D-xylo-4-hexulose-3-dehydrase
Cenrod_1819	signal transduction histidine kinase
Cenrod_1838	methyl-accepting chemotaxis protein
Cenrod_1881	methyl-accepting chemotaxis protein
Cenrod_1883	methyl-accepting chemotaxis protein WspA
Cenrod_1886	chemotaxis-related methylase
Cenrod_1887	signal transduction protein
Cenrod_1897	response regulator
Cenrod_1899	signal transduction histidine kinase
Cenrod_1919	beta-lactamase-like protein
Cenrod_1924	methyl-accepting chemotaxis protein
Cenrod_1931	response regulator-like protein
Cenrod_1933	transposase
Cenrod_1943	signal transduction protein
Cenrod_1956	retron-type reverse transcriptase
Cenrod_1961	retron-type reverse transcriptase
Cenrod_1966	retron-type reverse transcriptase
Cenrod_1972	transposase
Cenrod_1984	retron-type reverse transcriptase
Cenrod_1985	restriction endonuclease
Cenrod_1988	GGDEF domain protein
Cenrod_2030	chemotaxis protein CheZ
Cenrod_2032	HD-GYP domain protein
Cenrod_2033	CheY-like chemotaxis protein
Cenrod_2034	signal transduction histidine kinase
Cenrod_2036	ABC-type amino acid transporter substrate-binding protein
Cenrod_2068	methyltransferase type 11
Cenrod_2069	methyltransferase type 11
Cenrod_2071	transposase
Cenrod_2072	transposase
Cenrod_2073	glycosyltransferase
Cenrod_2075	transposase
Cenrod_2076	transposase
Cenrod_2081	methyltransferase FkbM
Cenrod_2086	dehydrogenase-like protein
Cenrod_2095	signal transduction histidine kinase

Cenrod_2097	type II secretory pathway protein
Cenrod_2098	general secretion pathway protein G
Cenrod_2099	general secretion pathway protein G
Cenrod_2114	DNA repair protein RadC
Cenrod_2116	bacteriophytochrome
Cenrod_2131	HD-GYP domain protein
Cenrod_2133	kinase-like protein
Cenrod_2134	chemotaxis protein CheW
Cenrod_2136	methyl-accepting chemotaxis protein
Cenrod_2137	chemotaxis response regulator CheB
Cenrod_2138	chemotaxis methyltransferase CheR
Cenrod_2139	chemotaxis histidine kinase-like protein
Cenrod_2143	response regulator receiver protein
Cenrod_2144	F420-non-reducing hydrogenase subunit G
Cenrod_2145	F420-non-reducing hydrogenase subunit A
Cenrod_2146	hydrogenase maturation protease
Cenrod_2147	heterodisulfide reductase subunit A
Cenrod_2148	F420-non-reducing hydrogenase iron-sulfur subunit D
Cenrod_2154	ABC-type amino acid transporter subunit
Cenrod_2160	transposase
Cenrod_2162	type II restriction enzyme
Cenrod_2174	integron/retron-type RNA-directed DNA polymerase
Cenrod_2209	integrase catalytic subunit
Cenrod_2211	transposase
Cenrod_2222	retron-type reverse transcriptase
Cenrod_2236	type III restriction protein res subunit
Cenrod_2240	ABC-type molybdate transporter substrate-binding protein
Cenrod_2241	methyl-accepting chemotaxis protein
Cenrod_2247	DNA modification methylase
Cenrod_2260	SAM-dependent methyltransferase
Cenrod_2262	transcriptional factor-like protein
Cenrod_2281	polysaccharide exporter
Cenrod_2286	SAM-dependent methyltransferase
Cenrod_2289	capsular polysaccharide transporter permease protein
Cenrod_2290	capsular polysaccharide transporter permease protein
Cenrod_2292	glycosyltransferase
Cenrod_2293	calcium-binding RTX toxin-like protein
Cenrod_2296	outer membrane protein
Cenrod_2324	kinase-like protein
Cenrod_2325	signal transduction histidine kinase
Cenrod_2327	FkbM-family methyltransferase
Cenrod_2328	ATPase-like protein
Cenrod_2336	signal transduction histidine kinase
Cenrod_2338	PAS/PAC domain protein
Cenrod_2340	transposase

Cenrod_2341	methionine synthase I subunit
Cenrod_2343	PilT domain protein
Cenrod_2344	transposase
Cenrod_2345	transposase
Cenrod_2352	transposase
Cenrod_2360	transposase
Cenrod_2361	transposase
Cenrod_2362	transposase
Cenrod_2363	group II intron maturase-specific domain protein
Cenrod_2365	transposase
Cenrod_2384	response regulator
Cenrod_2386	methyl-accepting chemotaxis protein
Cenrod_2388	surface protein SUR1
Cenrod_2390	glycosyltransferase
Cenrod_2391	cell wall biogenesis glycosyltransferase
Cenrod_2392	glycosyltransferase-like protein
Cenrod_2393	methyltransferase FkbM
Cenrod_2395	adenylate cyclase
Cenrod_2398	GGDEF domain protein
Cenrod_2416	prevent-host-death family protein
Cenrod_2417	PilT domain protein
Cenrod_2418	integron/retron-type RNA-directed DNA polymerase
Cenrod_2419	group II intron maturase-specific domain protein
Cenrod_2425	group II intron maturase-specific domain protein
Cenrod_2427	DNA replication protein
Cenrod_2430	virulence-related protein
Cenrod_2431	type I restriction enzyme subunit S
Cenrod_2432	type I restriction enzyme subunit S
Cenrod_2433	DNA-binding domain protein
Cenrod_2434	type I restriction-modification system methyltransferase subunit
Cenrod_2435	transposase
Cenrod_2436	type I restriction-modification system methyltransferase subunit
Cenrod_2444	integrase
Cenrod_2446	O-linked N-acetylglucosamine transferase-like protein
Cenrod_2447	outer membrane protein
Cenrod_2448	membrane protein
Cenrod_2449	ABC-type transporter component
Cenrod_2455	GGDEF domain protein
Cenrod_2475	signal transduction histidine kinase
Cenrod_2476	phosphonate transporter substrate-binding protein
Cenrod_2480	transposase
Cenrod_2493	SAM-dependent methyltransferase
Cenrod_2494	guanosine polyphosphate pyrophosphohydrolase/synthetase
Cenrod_2506	signal transduction histidine kinase
Cenrod_2507	general secretion pathway protein E

Cenrod_2509	ABC-type cobalt transporter permease protein
Cenrod_2520	long-chain fatty acid transport protein
Cenrod_2539	nucleic acid-binding-like protein
Cenrod_2540	type I restriction enzyme-like protein
Cenrod_2548	transposase
Cenrod_2569	cobalamin binding-like protein
Cenrod_2570	signal transduction histidine kinase HydH
Cenrod_2571	carbonic anhydrase
Cenrod_2578	signal transduction histidine kinase
Cenrod_2586	signal transduction histidine kinase
Cenrod_2588	signal transduction histidine kinase
Cenrod_2598	ATPase-like protein
Cenrod_2600	DNA methyltransferase-like protein
Cenrod_2601	DNA methylase
Cenrod_2621	c-di-GMP-specific phosphodiesterase
Cenrod_2622	signal transduction type protein
Cenrod_2629	phosphoserine aminotransferase
Cenrod_2633	PAS/PAC domain protein
Cenrod_2634	signal transduction histidine kinase
Cenrod_2636	signal transduction protein
Cenrod_2639	signal transduction histidine kinase
Cenrod_2640	signal transduction protein
Cenrod_2642	heme oxygenase
Cenrod_2646	phosphonate transporter substrate-binding protein
Cenrod_2647	signal transduction histidine kinase
Cenrod_2648	cell cycle response regulator
Cenrod_2657	spore maturation protein CgeD
Cenrod_2658	type 12 methyltransferase
Cenrod_2665	transposase
Cenrod_2666	DNA replication protein
Cenrod_2669	transposase
Cenrod_2673	dinucleotide-utilizing enzyme
Cenrod_2674	UBA/ThiF-type NAD/FAD-binding protein
Cenrod_2676	ATPase-like protein
Cenrod_2679	transposase
Cenrod_2680	transposase
Cenrod_2683	NTPase-like protein
Cenrod_2684	ATPase-like protein
Cenrod_2690	DNA-directed RNA polymerase I subunit A1
Cenrod_2692	transcriptional regulator
Cenrod_2693	thymidine phosphorylase
Cenrod_2700	cAMP-induced filamentation protein
Cenrod_2702	transposase
Cenrod_2703	type I restriction enzyme subunit M
Cenrod_2705	ATPase-like protein

Cenrod_2707	type I restriction enzyme subunit R
Cenrod_2708	ATP-dependent DNA helicase

Table S4. Potential horizontal transferred gene pairs of "*Ca.* S. mobilis" and *Chl. chlorochromatii*. Defined by higher similarity to each other than most, if not all, genes in databases. Annotations of genes in "*Ca.* S. mobilis" are listed here.

Locus tag in "*Ca.* S. mobilis"	Locus tag in *C. chlorochromatii*	Annotation
Cenrod_0015	Cag_0671	dTDP-glucose 4,6-dehydratase
Cenrod_0016	Cag_0672	pyridoxal phosphate-dependent enzyme
Cenrod_0017	Cag_0673	glycosyltransferase-like protein
Cenrod_1104	Cag_0293	Virulence related protein
Cenrod_1719	Cag_1343	hypothetical protein
Cenrod_1720	Cag_1342	hypothetical protein
Cenrod_1957	Cag_0305	hypothetical protein
Cenrod_1958	Cag_0304	hypothetical protein
Cenrod_2171	Cag_1177	hypothetical protein
Cenrod_2172	Cag_1178	hypothetical protein
Cenrod_2220	Cag_0276	hypothetical protein
Cenrod_2335	Cag_0747	hypothetical protein
Cenrod_2562	Cag_0721	hypothetical protein
